# Comparative analyses of chloroplast genomes in *Geum* species: insights into genome characteristics, phylogenomic implications, and adaptive evolution

**DOI:** 10.3389/fpls.2025.1713809

**Published:** 2025-12-04

**Authors:** Wen-Tao Fu, Zhi-Ping Zhang, Jia-Jie Guo, Jun Wen, Qin-Qin Li

**Affiliations:** 1 College of Life Science and Technology, Inner Mongolia Normal University, Hohhot, China; 2 Key Laboratory of Biodiversity Conservation and Sustainable Utilization in Mongolian Plateau for College and University of Inner Mongolia Autonomous Region, Hohhot, China; 3 College of Computer Science and Technology, Inner Mongolia Normal University, Hohhot, China; 4 Department of Botany, National Museum of Natural History, Smithsonian Institution, Washington, DC, United States

**Keywords:** *Geum*, chloroplast genome, comparative analyses, phylogeny, adaptive evolution

## Abstract

The genus *Geum*, comprises about 72 species, most frequently distributed in North America, Asia, and Europe, with a few representatives in South America, South Africa, Australia, and New Zealand. Previous phylogenetic analyses based on several molecular markers have contributed to understanding the delimitation of *Geum*, but the phylogenetic relationships within the genus remain unresolved. Moreover, only a few chloroplast (cp) genomes of *Geum* species have been reported, and no comparative cp genome analyses among *Geum* species have been conducted to date, limiting our understanding of cp genome evolution. This study is the first to conduct comparative genomic analyses on the cp genomes of 32 accessions representing 11 *Geum* taxa. The *Geum* cp genomes showed a typical quadripartite structure, similar to that of most other land plants, with a total of 129 genes, including 84 protein-coding genes (PCGs), 37 transfer RNA (tRNA) genes, and eight ribosomal RNA (rRNA) genes. The *Geum* cp genomes were conserved in structure, size, GC content, gene order, and gene content. Eleven highly variable regions (*3′-trnK-UUU-matK*, *psbZ-trnG-GCC*, *trnR-UCU-atpA*, *petA-psbJ*, *5′-trnK-UUU-rps16*, *rps16-trnQ-UUG*, *rpl32-trnL-UAG*, *ndhF-rpl32*, *trnS-GCU-trnG-UCC*, *ndhC-trnV-UAC*, and *petN-psbM*) were identified as candidate molecular markers for future studies on population genetics and systematic evolution of *Geum* species. Phylogenetic analyses provided new insights into the relationships among *Geum* species and supported Smedmark’s recircumscription of *Geum* in a broad sense, corroborating the inclusion of *Acomastylis*, *Coluria*, and *Taihangia* within *Geum*. Twenty-three genes with sites under positive selection were detected, and the adaptive evolution of these genes may play important roles in the adaptation of *Geum* species to their habitats. Overall, this study enhances our understanding of the cp genome characteristics, phylogeny, and adaptive evolution of *Geum* species.

## 1 Introduction

The chloroplast (cp) is a semiautonomous organelle in green plants that plays key roles in photosynthesis and other aspects of plant physiology and development (Neuhaus and Emes, 2000; Sato et al., 2003; Daniell et al., 2016). The cp genomes of land plants are circular, double-stranded molecules, mostly ranging from 115 to 165 kb in size and containing 120–130 genes (Ravi et al., 2008; Daniell et al., 2016). Land plant cp genomes usually display a typical quadripartite structure, with a large single-copy region (LSC) plus a small single-copy region (SSC) separated by two inverted repeat regions, IRa and IRb (Ravi et al., 2008; Wicke et al., 2011; Daniell et al., 2016). Owing to their small size, moderate substitution rate, conserved structure, and lack of recombination (Palmer, 1985; Drouin et al., 2008; Ravi et al., 2008; Mower and Vickrey, 2018), cp genomes have become important tools for studies on species identification, population genetics, taxonomy, biogeography, and systematic evolution in land plants, particularly with the development of high-throughput sequencing technology (e.g., Li et al., 2021; Wang et al., 2022; Zhang G. J. et al., 2022; Hu et al., 2023; Li et al., 2024a; Zhou et al., 2024; Rana et al., 2025).

As the largest genus, comprising about 72 of the 75 species in its tribe, *Geum* in the sense of Smedmark (2006), together with the other two woody and white-flowered genera *Fallugia* and *Sieversia*, constitutes the tribe Colurieae (Smedmark and Eriksson, 2002; Smedmark et al., 2003; Smedmark, 2006). From a geographical perspective, the monospecific *Fallugia* occurs in the southwestern USA and northern Mexico, *Sieversia* occurs in Alaska and East Asia, and species of *Geum* are most frequently distributed in North America, Asia, and Europe, with a few representatives in South America, South Africa, Australia, and New Zealand (Gajewski, 1959; Smedmark and Eriksson, 2002; Brouillet, 2014; Henrickson and Parfitt, 2014; Phipps, 2014; Rohrer, 2014). In addition to their ornamental value, the primary importance of *Geum* species lies in their medicinal properties. Some *Geum* species have been used in traditional medicine for the treatment of various conditions, including leucorrhea, hemorrhages, gingivitis, muscle pain, gastrointestinal disorders, cardiac disorders, infections, fever, and inflammation of the skin, mucous membranes, and urinary system (Hänsel et al., 1993; Vollmann and Scbultze, 1995; Birnesser and Stolt, 2007; Redžić, 2007; Menković et al., 2011; Granica et al., 2016; Blaschek et al., 2018). The classification of *Geum*, which over the last century was mainly based on morphological evidence, cytogenetic studies, and interspecific crossings, has been ambiguous and conflicting (e.g., Scheutz, 1870; Focke, 1894; Rydberg, 1913; Bolle, 1933; Juzepchuk, 1941; Gajewski, 1957, 1959, 1968; Schulze-Menz, 1964; Hutchinson, 1967; Kalkman, 1988). Later molecular phylogenetic studies based on the cp *trnL*-*trnF* region and nuclear ribosomal ITS (Smedmark and Eriksson, 2002), as well as the low-copy nuclear gene GBSSI (Smedmark et al., 2003, 2005), did not support the monophyly of any of the previously proposed circumscriptions of *Geum*, and provided good support for delimiting the herbaceous perennials with a rosette of basal leaves in the tribe Colurieae as *Geum* in a broad sense (Smedmark, 2006). *Geum*, with this broad recircumscription (sensu Smedmark, 2006), embraces 12 historically segregated genera, namely *Waldsteinia*, *Stylypus*, *Coluria*, *Acomastylis*, *Erythrocoma*, *Novosieversia*, *Oncostylus*, *Parageum*, *Orthurus*, *Woronowia*, *Taihangia*, and *Oreogeum*. Previous phylogenetic analyses based on several molecular markers have contributed to understanding the delimitation of *Geum*, but the phylogenetic relationships within the genus remain unresolved (Smedmark and Eriksson, 2002; Smedmark et al., 2003, 2005; Faghir et al., 2018). Moreover, only a few cp genomes of *Geum* species have been reported (e.g., Li and Wen, 2021; Feng et al., 2022; Guo et al., 2023), and, to the best of our knowledge, no comparative cp genome analyses among *Geum* species have been conducted to date, limiting our understanding of cp genome evolution in this genus.

This is the first study to conduct comparative genomic analyses of the cp genomes of 32 accessions representing 11 *Geum* taxa. The aims were to (1) analyze the cp genome characteristics of *Geum* species to explore its cp genome evolution, (2) identify mutational hotspot regions across the cp genomes of *Geum* as potential molecular markers for species identification and phylogenetic studies, (3) provide insights into the phylogenetic relationships among *Geum* species to enhance understanding of their classification, and (4) investigate the adaptive evolution of cp genes in *Geum* species to understand their molecular adaptation. This study lays a foundation for future research on molecular identification, phylogenetics, and cp genome evolution of *Geum* species, and also provides an important theoretical basis for the development and utilization of the medicinal plant resources of *Geum*.

## 2 Materials and methods

### 2.1 Taxon sampling, DNA extraction, and Illumina sequencing

A total of 32 cp genome sequences representing 11 *Geum* taxa (17 of which were newly sequenced) were sampled in this study. The 17 new samples of *Geum* were collected during field trips, and species identification of the collected samples was conducted using an optical microscope with reference to relevant literature (Yü and Kuan, 1985; Yü and Li, 1985; Li et al., 2003). Voucher specimen information and GenBank accession numbers for *Geum* samples are presented in **Table 1**. In addition, cp genome sequences of *Fallugia paradoxa*, *Potentilla suavis*, *Rosa multiflora*, *Agrimonia pilosa*, and *Rubus alceifolius* downloaded from GenBank were included in the phylogenetic analyses, following Zhang et al. (2017). Total genomic DNA was isolated from silica-dried leaves using the CTAB method (Doyle and Doyle, 1987). Sonication was then used to fragment the DNA, and the DNA fragments were used to construct short-insert libraries with an insert size of 300 bp. Finally, the pooled libraries were sequenced on the Illumina NovaSeq platform in Novogene (Beijing, China).

**Table 1:** Summary of voucher specimens and chloroplast genome characteristics for *Geum* species.

Species name (synonym)	Voucher	Locality	GenBank accession	Size (bp)				Number of genes				GC content (%)				References
				Total	LSC	SSC	IR	Total	Protein coding	tRNA	rRNA	Total	LSC	SSC	IR	
*Geum aleppicum* Jacq. 1			OK509085	156,036	85,358	18,410	26,134	129	84 (6)	37 (7)	8 (4)	36.8	34.4	30.7	42.6	Zhang P. et al. (2022)
*Geum aleppicum* Jacq. 2			OM461318	155,911	85,382	18,289	26,120	129	84 (6)	37 (7)	8 (4)	36.8	34.4	30.7	42.7	Unpublished
*Geum aleppicum* Jacq. 3	Li QQ 20220722006 (NMTC)	China, Jilin, Antu	PX414088	155,940	85,370	18,320	26,125	129	84 (6)	37 (7)	8 (4)	36.8	34.4	30.7	42.7	This article
*Geum aleppicum* Jacq. 4	Li QQ 20220716002 (NMTC)	China, Sichuan, Daofu	PX414089	156,038	85,362	18,418	26,129	129	84 (6)	37 (7)	8 (4)	36.8	34.5	30.7	42.6	This article
*Geum aleppicum* Jacq. 5	Li QQ 20220812064 (NMTC)	China, Inner Mongolia, Hohhot	PX414090	156,038	85,362	18,418	26,129	129	84 (6)	37 (7)	8 (4)	36.8	34.5	30.7	42.6	This article
*Geum aleppicum* Jacq. 6	Li QQ 20160717001 (NMTC)	China, Xinjiang, Fuyun	PX414091	155,943	85,373	18,320	26,125	129	84 (6)	37 (7)	8 (4)	36.8	34.4	30.8	42.7	This article
*Geum elatum* Wall. ex G.Don var. *elatum* 1 [*Acomastylis elata* (Wall. ex G.Don) F.Bolle var. *elata*]	Li QQ 20150822024 (NMTC)	China, Sichuan, Dege	PX414092	156,114	85,452	18,402	26,130	129	84 (6)	37 (7)	8 (4)	36.7	34.3	30.8	42.6	This article
*Geum elatum* Wall. ex G.Don var. *elatum* 2 [*Acomastylis elata* (Wall. ex G.Don) F.Bolle var. *elata*]			MT982432	156,104	85,507	18,501	26,048	129	84 (6)	37 (7)	8 (4)	36.7	34.3	30.8	42.7	Unpublished
*Geum elatum* Wall. ex G.Don var. *elatum* 3 [*Acomastylis elata* (Wall. ex G.Don) F.Bolle var. *elata*]	Li QQ 20150729073 (NMTC)	China, Sichuan, Yajiang	PX414093	156,145	85,544	18,509	26,046	129	84 (6)	37 (7)	8 (4)	36.7	34.3	30.8	42.7	This article
*Geum elatum* Wall. ex G.Don var. *elatum* 4 [*Acomastylis elata* (Wall. ex G.Don) F.Bolle var. *elata*]	Li QQ 20230720004 (NMTC)	China, Xizang, Yadong	PX414094	156,248	85,610	18,530	26,054	129	84 (6)	37 (7)	8 (4)	36.7	34.3	30.8	42.7	This article
*Geum elatum* Wall. ex G.Don var. *elatum* 5 [*Acomastylis elata* (Wall. ex G.Don) F.Bolle var. *elata*]			KY419976													Zhang et al. (2017)
*Geum elatum* Wall. ex G.Don var. *humile* (Royle) Hook.f. [*Acomastylis elata* (Wall. ex G.Don) F.Bolle var. *humilis* (Royle) F.Bolle]	Li QQ LWQ0819002 (NMTC)	China, Xizang, Leiwuqi	PX414095	156,121	85,508	18,501	26,056	129	84 (6)	37 (7)	8 (4)	36.7	34.3	30.8	42.7	This article
*Geum henryi* (Batalin) Smedmark 1 [*Coluria henryi* Batalin]	Li QQ 20220717008 (NMTC)	China, Sichuan, Guangwu Mountain	PX414096	155,175	85,563	18,466	25,573	129	84 (6)	37 (7)	8 (4)	36.7	34.3	30.7	42.8	This article
*Geum henryi* (Batalin) Smedmark 2 [*Coluria henryi* Batalin]	Li QQ 20220718002 (NMTC)	China, Sichuan, Micang Mountain	PX414097	155,291	85,740	18,419	25,566	129	84 (6)	37 (7)	8 (4)	36.7	34.3	30.8	42.8	This article
*Geum japonicum* Thunb. var. *chinense* F.Bolle 1			MW770453	155,999	85,333	18,410	26,128	129	84 (6)	37 (7)	8 (4)	36.8	34.4	30.7	42.6	Unpublished
*Geum japonicum* Thunb. var. *chinense* F.Bolle 2			MW770454	155,912	85,375	18,297	26,120	129	84 (6)	37 (7)	8 (4)	36.8	34.4	30.7	42.7	Unpublished
*Geum japonicum* Thunb. var. *chinense* F.Bolle 3	Li QQ 20220711012 (NMTC)	China, Sichuan, Dayi	PX414098	156,007	85,340	18,411	26,128	129	84 (6)	37 (7)	8 (4)	36.8	34.5	30.7	42.6	This article
*Geum japonicum* Thunb. var. *chinense* F.Bolle 4	Li QQ 20220709002 (NMTC)	China, Sichuan, Dujiangyan	PX414099	156,009	85,342	18,411	26,128	129	84 (6)	37 (7)	8 (4)	36.8	34.4	30.7	42.6	This article
*Geum japonicum* Thunb. var. *chinense* F.Bolle 5	Li QQ 20220713001 (NMTC)	China, Sichuan, Mount Emei	PX414100	156,013	85,347	18,410	26,128	129	84 (6)	37 (7)	8 (4)	36.8	34.4	30.7	42.6	This article
*Geum japonicum* Thunb. var. *chinense* F.Bolle 6	Li QQ 20160807032 (NMTC)	China, Yunnan, Luquan	PX414101	155,856	85,375	18,271	26,105	129	84 (6)	37 (7)	8 (4)	36.8	34.4	30.8	42.7	This article
*Geum japonicum* Thunb. var. *chinense* F.Bolle 7	Li QQ 20150728012 (NMTC)	China, Sichuan, Luding	PX414102	155,831	85,350	18,271	26,105	129	84 (6)	37 (7)	8 (4)	36.8	34.4	30.8	42.7	This article
*Geum longifolium* (Maxim.) Smedmark [*Coluria longifolia* Maxim.]			OP161499	155,884	85,338	18,358	26,094	129	84 (6)	37 (7)	8 (4)	36.7	34.4	30.9	42.6	Guo et al. (2023)
*Geum macrophyllum* Willd.			MT774132	155,940	85,307	18,329	26,152	129	84 (6)	37 (7)	8 (4)	36.6	34.3	30.6	42.6	Li and Wen (2021)
*Geum omeiense* (T.C.Ku) Smedmark 1 [*Coluria omeiensis* T.C.Ku]	LCH 1146 (NMTC)	China, Sichuan, Mount Emei	PX414103	155,388	85,538	18,140	25,855	129	84 (6)	37 (7)	8 (4)	36.6	34.3	30.8	42.6	This article
*Geum omeiense* (T.C.Ku) Smedmark 2 [*Coluria omeiensis* T.C.Ku]	LCH 1148 (NMTC)	China, Sichuan, Mount Emei	PX414104	155,388	85,538	18,140	25,855	129	84 (6)	37 (7)	8 (4)	36.6	34.3	30.8	42.6	This article
*Geum rupestre* (T.T.Yü & C.L.Li) Smedmark 1 [*Taihangia rupestris* T.T.Yü & C.L.Li]			MZ151697	155,558	85,857	18,543	25,579	129	84 (6)	37 (7)	8 (4)	36.8	34.5	30.8	42.8	Feng et al. (2022)
*Geum rupestre* (T.T.Yü & C.L.Li) Smedmark 2 [*Taihangia rupestris* T.T.Yü & C.L.Li]			MG262388	155,479	85,771	18,550	25,579	129	84 (6)	37 (7)	8 (4)	36.8	34.5	30.8	42.8	Duan et al. (2018)
*Geum rupestre* (T.T.Yü & C.L.Li) Smedmark 3 [*Taihangia rupestris* T.T.Yü & C.L.Li]			ON873898	155,514	85,823	18,533	25,579	129	84 (6)	37 (7)	8 (4)	36.8	34.5	30.8	42.8	Liu et al. (2025)
*Geum rupestre* (T.T.Yü & C.L.Li) Smedmark 4 [*Taihangia rupestris* T.T.Yü & C.L.Li]			ON873891	155,512	85,838	18,496	25,589	129	84 (6)	37 (7)	8 (4)	36.8	34.5	30.9	42.8	Liu et al. (2025)
*Geum triflorum* Pursh			KY419977													Zhang et al. (2017)
*Geum urbanum* L. 1			OX327019													Unpublished
*Geum urbanum* L. 2			ON556622													Unpublished

### 2.2 Chloroplast genome assembly and annotation

Illumina paired-end sequencing generated about 5.0 Gb of raw data for each *Geum* sample. Adapters were removed from the raw reads using Trimmomatic (Bolger et al., 2014). NOVOPlasty (Dierckxsens et al., 2017) was employed to assemble the newly sequenced cp genomes from the filtered reads. During assembly, the cp genome of *Geum macrophyllum* (GenBank Accession No. MT774132) was used as the reference sequence, with its *rbcL* gene as seed input, and all other parameters set to default. After successfully assembling the cp genome sequences of some species, these sequences were used as references to assemble cp genomes of other accessions or closely related species. Using the cp genome of *G. macrophyllum* (MT774132) as the reference, cp genome annotations of *Geum* species downloaded from GeneBank were checked, and the cp genome sequence of *Geum elatum* (MT982432) was annotated by transfer annotation in Geneious Prime (Kearse et al., 2012). For newly sequenced *Geum* cp genome sequences, annotated sequences of the same species or closely related species were selected for transfer annotation.

### 2.3 Comparative analyses of chloroplast genomes

Comparative analyses were conducted on the complete cp genomes of 28 accessions representing *Geum* taxa. The whole cp genome size, lengths of the LSC/SSC/IR, Guanine-Cytosine (GC) content, gene composition, and boundary region variation were analyzed in Geneious Prime, and the variation in the LSC/IR/SSC boundary regions was illustrated. The cp genomes of *Geum* were aligned using MAUVE v. 2.4.0 (Darling et al., 2004, 2010) to identify potential rearrangements and inversions. The level of differentiation among the *Geum* cp genomes was assessed using the Shuffle-LAGAN mode in mVISTA (Frazer et al., 2004) with *Geum aleppicum* 1 as the reference. Coding and noncoding regions of the *Geum* cp genomes were extracted in Geneious Prime, and homologous loci were then aligned by MAFFT v. 7.490 (Katoh and Standley, 2013). Nucleotide variability (Pi) of each region was calculated using DnaSP v. 6.12.03 (Rozas et al., 2017). Both sequence lengths and Pi values were considered to screen candidate molecular markers for *Geum*. A tree-based method was further employed to evaluate the resolution power of the screened candidate molecular markers compared to the core DNA barcodes (*trnH-GUG-psbA*, *rbcL*, and *matK*). MEGA v. 12.0.11 (Kumar et al., 2024) was used to construct neighbor-joining (NJ) trees based on each molecular marker, using the “pairwise deletion” option to treat gaps/missing data and the “d: Transitions + Transversions” option for substitutions under the Kimura 2-parameter model, with 1,000 bootstrap replicates.

### 2.4 Phylogenetic analyses

Maximum likelihood (ML) and Bayesian inference (BI) methods were used to infer the phylogenetic relationships of the 11 *Geum* taxa within the phylogenetic framework of the tribe Colurieae. Based on previous studies (Zhang et al., 2017; Xiang et al., 2017), *Agrimonia pilosa*, *Potentilla suavis*, *Rosa minutifolia*, and *Rubus alceifolius* were selected as outgroups. A total of 37 cp genome sequences, with the IRa removed, were used for phylogenetic analyses (**Supplementary Table S1**), and these sequences were first aligned using MAFFT v. 7.490 (Katoh and Standley, 2013). The alignment was then trimmed using trimAL v. 1.4 (Capella-Gutiérrez et al., 2009) with a 0.75 gap threshold. RAxML v. 8.2.12 (Stamatakis, 2014) was employed to conduct the ML analysis under the GTRGAMMA model with 1,000 bootstrap replicates. Prior to the BI analysis, the best-fit model was selected using PartitionFinder2 (Lanfear et al., 2017) according to the Corrected Akaike Information Criterion (AICc; Sugiura, 1978) following Posada and Buckley (2004). The BI analysis was then performed using MrBayes v. 3.2.7a (Ronquist et al., 2012) under the best-fit model GTR + I + G. Markov Chain Monte Carlo (MCMC) analyses included four parallel runs with one million generations, sampled every 100 generations, with the initial 25% of trees discarded as burn-in, and the remaining trees used to generate a consensus tree.

### 2.5 Adaptive evolution analyses

Selection pressure analyses were conducted using CodeML (Yang, 2007) implemented in EasyCodeML (Gao et al., 2019), involving 32 complete cp genomes representing nine *Geum* taxa (28 accessions) and four related species (*Potentilla suavis*, *Rosa multiflora*, *Agrimonia pilosa*, *Rubus alceifolius*). First, the 78 common protein-coding genes (PCGs) were extracted from the 32 cp genomes using Geneious Prime (Kearse et al., 2012). Each PCG was then aligned separately by codons using MAFFT, with stop codons manually removed from each alignment. The alignments of the 78 PCGs were concatenated into a supermatrix for subsequent analysis. The FASTA format of the supermatrix was used as the input file in EasyCodeML. The ML tree established based on the supermatrix by RAxML v. 8.2.12 (Stamatakis, 2014), using the same parameters in the phylogenetic analyses, was used as an input tree (**Supplementary Figure S1**). The likelihood ratio test (LRT) was performed to detect adaptation signatures under four comparison site models: M0 (one-ratio) *vs*. M3 (discrete), M1a (neutral) *vs*. M2a (positive selection), M7 (beta) *vs*. M8 (beta and *ω* > 1), and M8a (beta and *ω* = 1) *vs*. M8, with significance threshold of *p* < 0.05. Bayesian empirical Bayes (BEB) (Yang et al., 2005) or Naïve empirical Bayes (NEB) (Nielsen and Yang, 1998) analysis was performed to detect sites under positive selection with posterior probabilities ≥ 0.95.

## 3 Results and discussion

### 3.1 Chloroplast genome characteristics

**Figure 1:**
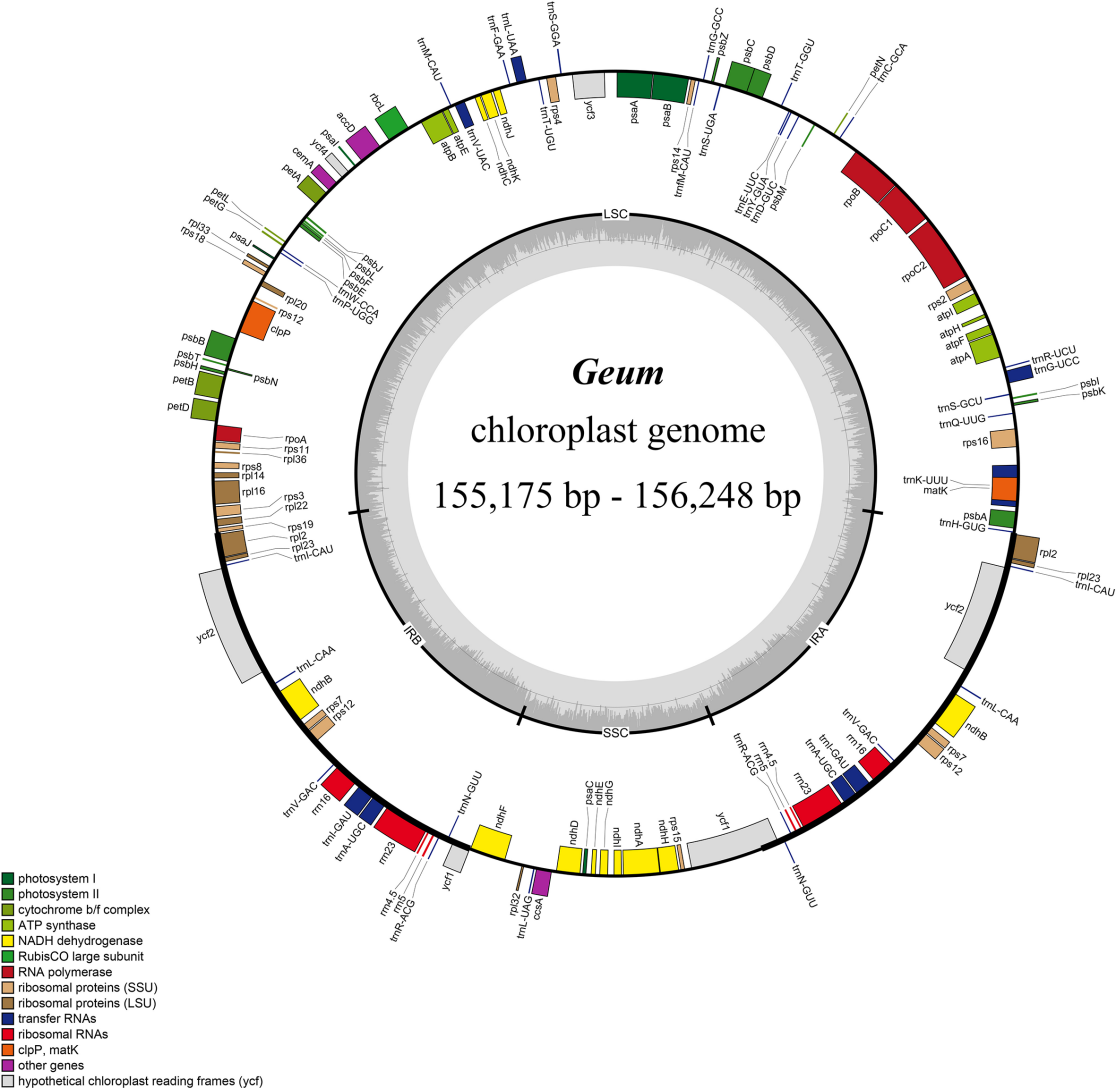
Chloroplast genome map of *Geum* species. Genes inside the circle are transcribed clockwise, and those outside are transcribed counterclockwise.

The size of the 28 *Geum* cp genomes ranged from 155,175 bp (*Geum henryi* 1) to 156,248 bp (*G. elatum* 4) (**Table 1**; **Figure 1**). The genomes exhibited a typical quadripartite structure, as observed in most land plants (Daniell et al., 2016), comprising an LSC region of 85,307 bp (*G. macrophyllum*) to 85,857 bp (*Geum rupestre* 1), an SSC region of 18,140 bp (*Geum omeiense*) to 18,550 bp (*G. rupestre* 2), and a pair of IR regions of 25,566 bp (*G. henryi* 2) to 26,152 bp (*G. macrophyllum*). The total GC content of the 28 *Geum* cp genomes ranged from 36.6% to 36.8%, with the IR regions (42.6%–42.8%) showing higher GC content than the LSC (34.3%–34.5%) and SSC (30.7%–30.9%) regions (**Table 1**), likely due to the presence of ribosomal RNA (rRNA) genes (Ravi et al., 2008). All 28 *Geum* cp genomes encoded 129 genes, including 112 unique genes and 17 duplicated genes. The 112 unique genes consisted of 78 PCGs, 30 transfer RNA (tRNA) genes, and four rRNA genes (**Figure 1**; **Table 1**; **Supplementary Table S2**). The 17 genes duplicated in the IR regions comprised seven tRNA genes (*trnA-UGC*, *trnI-CAU*, *trnI-GAU*, *trnL-CAA*, *trnN-GUU*, *trnR-ACG*, and *trnV-GAC*), six PCGs (*ndhB*, *rpl2*, *rpl23*, *rps7*, *rps12*, and *ycf2*), and four rRNA genes (*rrn4.5*, *rrn5*, *rrn16*, and *rrn23*). Among the 17 genes containing introns, three genes (*clpP*, *rps12*, and *ycf3*) had two introns, while the remaining 14 genes (*ndhA*, *ndhB*, *petB*, *petD*, *rpl2*, *rpl16*, *rpoC1*, *rps16*, *trnA-UGC*, *trnG-UCC*, *trnI-GAU*, *trnK-UUU*, *trnL-UAA*, and *trnV-UAC*) each contained a single intron (**Supplementary Table S2**).

**Figure 2:**
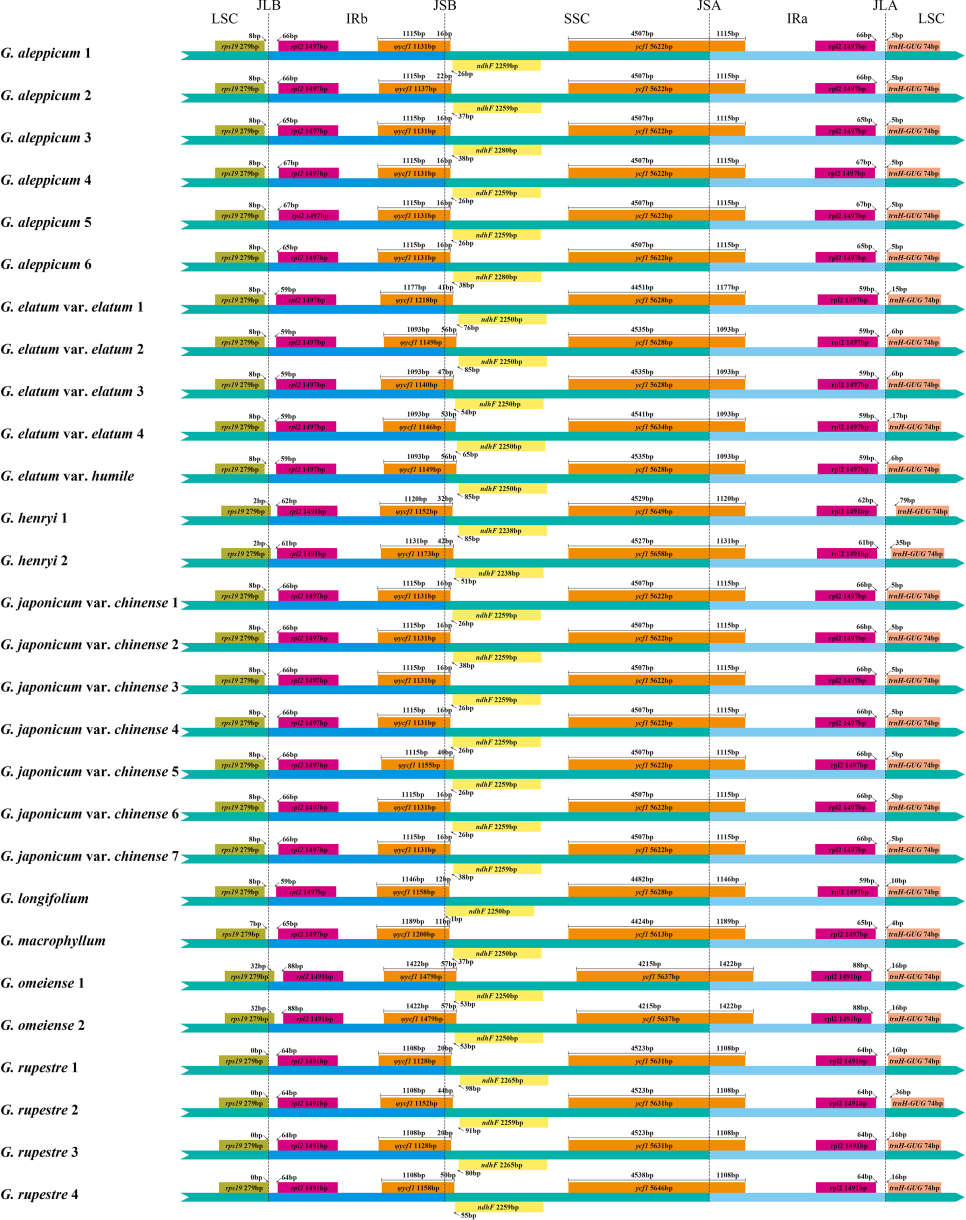
Comparison of the boundary regions of the large single-copy (LSC), small single-copy (SSC), and inverted repeat (IR) regions among 28 *Geum* chloroplast genomes.

No gene rearrangements or inversions were detected in the 28 *Geum* cp genomes based on Mauve alignment analysis, indicating strong collinearity among these genomes. Although the *Geum* cp genomes are highly conserved in gene content, organization, and order, minor variations were visible in the IR/SC boundary regions (**Figure 2**). Expansion and contraction of the IR regions are the primary drivers of cp genome size variation in terrestrial plants (Ravi et al., 2008; Mower and Vickrey, 2018). All 28 *Geum* cp genomes contained identical genes and pseudogenes at the boundary regions, including *rps19*, *rpl2*, *ѱycf1, ndhF*, *ycf1*, and *trnH-GUG.* The *rps19*-*rpl2*-*trnH-GUG* genes were located in the LSC/IR boundary regions. In *G. henryi* and *G. omeiense*, *rps19* crossed the LSC/IRb junction (JLB), extending 2 and 32 bp in the IRb region, respectively, whereas in the other taxa, *rps19* was entirely located in the LSC region, 0–8 bp from the JLB. The duplicated *rpl2* gene was located in both IRb and IRa regions, 59–88 bp away from the JLB and IRa/LSC junction (JLA), respectively. Gene *trnH-GUG* was positioned in the LSC region, 4–79 bp from the JLA. The pseudogene *ψycf1* and the *ndhF* gene were located around the IRb/SSC junction (JSB). The *ψycf1* pseudogene spanned the JSB, extending 11–56 bp into the SSC region, whereas *ndhF* was located in the SSC region, with 1–98 bp from the JSB region. In *Geum longifolium*, *G. japonicum* var. *chinense* 5, and *G. omeiense*, *ψycf1* overlapped with the *ndhF* gene by 4–14 bp. The *ycf1* gene crossed the SSC/IRa junction (JSA), with a length of 4,215–4,542 bp in the SSC region and 1,093–1,422 bp in the IRa region. The results indicated no significant expansion or contraction of the IR region in *Geum* cp genomes, supporting minor IR boundary shifts among closely related species (Shen et al., 2022; Li et al., 2024b; Jiang et al., 2025).

The mVISTA analysis revealed that *Geum* cp genomes were generally conserved at the genome-wide level, although several highly divergent regions were identified (**Supplementary Figure S2**). Overall, the LSC and SSC regions exhibited greater divergence than the IR regions. Noncoding regions, particularly intergenic spacers (IGS), were more variable than coding regions, consistent with observations in other angiosperms (e.g., Wu et al., 2020; Xu et al., 2021; Hang et al., 2025).

### 3.2 Divergence hotspots

**Figure 3:**
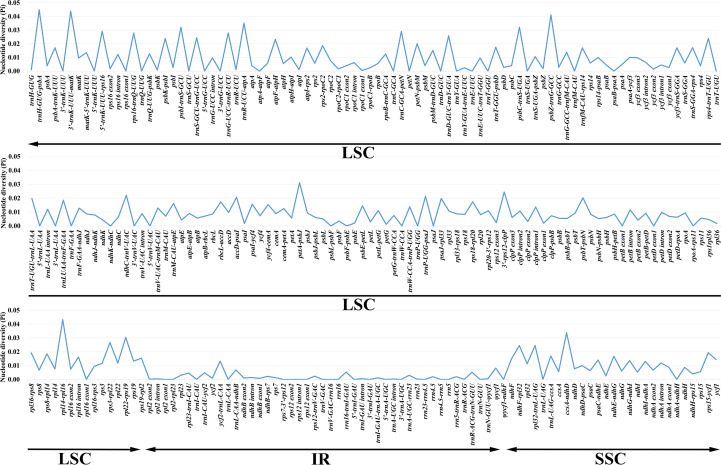
Comparison of the nucleotide diversity (Pi) values of 264 regions in 28 *Geum* chloroplast genomes.

Nucleotide variability (Pi) values for 264 regions in the 28 *Geum* cp genomes were analyzed using DnaSP v.6.12.03 (Rozas et al., 2017). Pi values ranged from 0 to 0.04487, with an average of 0.00843, indicating high similarity among *Geum* cp genomes (**Figure 3**; **Supplementary Table S3**). Four regions had Pi > 0.04, six regions had 0.03 < Pi ≤ 0.04, 18 regions had 0.02 < Pi ≤ 0.03, 53 regions had 0.01 < Pi ≤ 0.02, 128 regions had 0 < Pi ≤ 0.01, and 55 regions had Pi = 0. The four highly variable regions with Pi > 0.04 (*trnH*-*GUG-psbA*, *3′-trnK-UUU-matK*, *rpl14-rpl16*, and *psbZ-trnG-GCC*) were all located in the LSC region. Among the six regions with 0.03 < Pi ≤ 0.04 (*trnR-UCU-atpA*, *ccsA-ndhD*, *psbI-trnS-GCU*, *psbC-trnS-UGA*, *petA-psbJ*, and *rpl22-rps19*), *ccsA-ndhD* was in the SSC region, while the other five regions were in the LSC region. Of the 18 regions with 0.02 < Pi ≤ 0.03 (*5′-trnK-UUU-rps16*, *trnC-GCA-petN*, *trnG-UCC-trnR-UCU*, *rps16-trnQ-UUG*, *rps3-rpl22*, *trnD-GUC-trnY-GUA*, *rpl32-trnL-UAG*, *ndhF-rpl32*, *3′-rps12-clpP*, *trnS-GCU-trnG-UCC*, *rps4-trnT-UGU*, *psbK-psbI*, *atpF-atpH*, *ndhC-trnV-UAC*, *trnP-UGG-psaJ*, *accD-psaI*, *psbT-psbN*, and *petN-psbM*), two regions (*rpl32-trnL-UAG*, *ndhF-rpl32*) were in the SSC region, and the remaining 16 regions were located in the LSC region. In general, regions located in the IR region exhibited lower Pi values compared with those in the LSC and SSC regions, indicating that the IR region is relatively more conserved. Moreover, coding regions were less variable than noncoding regions, with the most highly variable regions located in the intergenic spacers.

Considering both sequence length and variability, among the 16 regions with Pi > 2% and alignment lengths > 400 bp, 11 regions (*3′-trnK-UUU-matK*, *psbZ-trnG-GCC*, *trnR-UCU-atpA*, *petA-psbJ*, *5′-trnK-UUU-rps16*, *rps16-trnQ-UUG*, *rpl32-trnL-UAG*, *ndhF-rpl32*, *trnS-GCU-trnG-UCC*, *ndhC-trnV-UAC*, and *petN-psbM*) were proposed as candidate molecular markers for *Geum*, suitable for developing specific DNA barcodes. In our study, the core DNA barcodes *trnH-GUG-psbA* exhibited the highest Pi value of 0.04487, whereas *matK* and *rbcL* showed relatively low Pi values of 0.00962 and 0.00869, respectively. Although *trnH-GUG-psbA* exhibited the highest Pi value, its relatively short length limits the number of informative sites. The cp molecular markers *trnL* intron and *trnL*-*trnF* intergenic spacer, previously used in phylogenetic studies of *Geum* species (Smedmark and Eriksson, 2002; Faghir et al., 2018; Lv et al., 2020; Protopopova et al., 2023), had Pi values of only 0.01198 and 0.01856 in our dataset, respectively. To further assess the resolving power of the 11 candidate molecular markers compared with the core DNA barcodes, NJ trees were reconstructed individually for each sequence (**Supplementary Figures S3–S16**). The resolution ability of these sequences was evaluated based on both the number of successfully identified species and the support values in the NJ tree. Overall, the 11 candidate molecular markers demonstrated better resolution than the core DNA barcodes *trnH-GUG-psbA* and *rbcL*. Among the 11 candidates, nine markers—excluding *psbZ-trnG-GCC* and *5′-trnK-UUU-rps16*—also outperformed the core DNA barcode *matK*. In this study, the relatively lower resolution of *psbZ-trnG-GCC* and *5′-trnK-UUU-rps16* compared with *matK* was mainly due to their inability to correctly identify *G. henryi*. In future research, the utility of the 11 candidate molecular markers for *Geum* can be further evaluated with more detailed taxon sampling and larger numbers of population samples per species. In conclusion, the development of specific molecular markers for particular taxonomic groups is necessary, and the new candidate markers identified in this study will facilitate future research on species identification and the phylogeny of *Geum*.

### 3.3 Phylogenetic analyses

**Figure 4:**
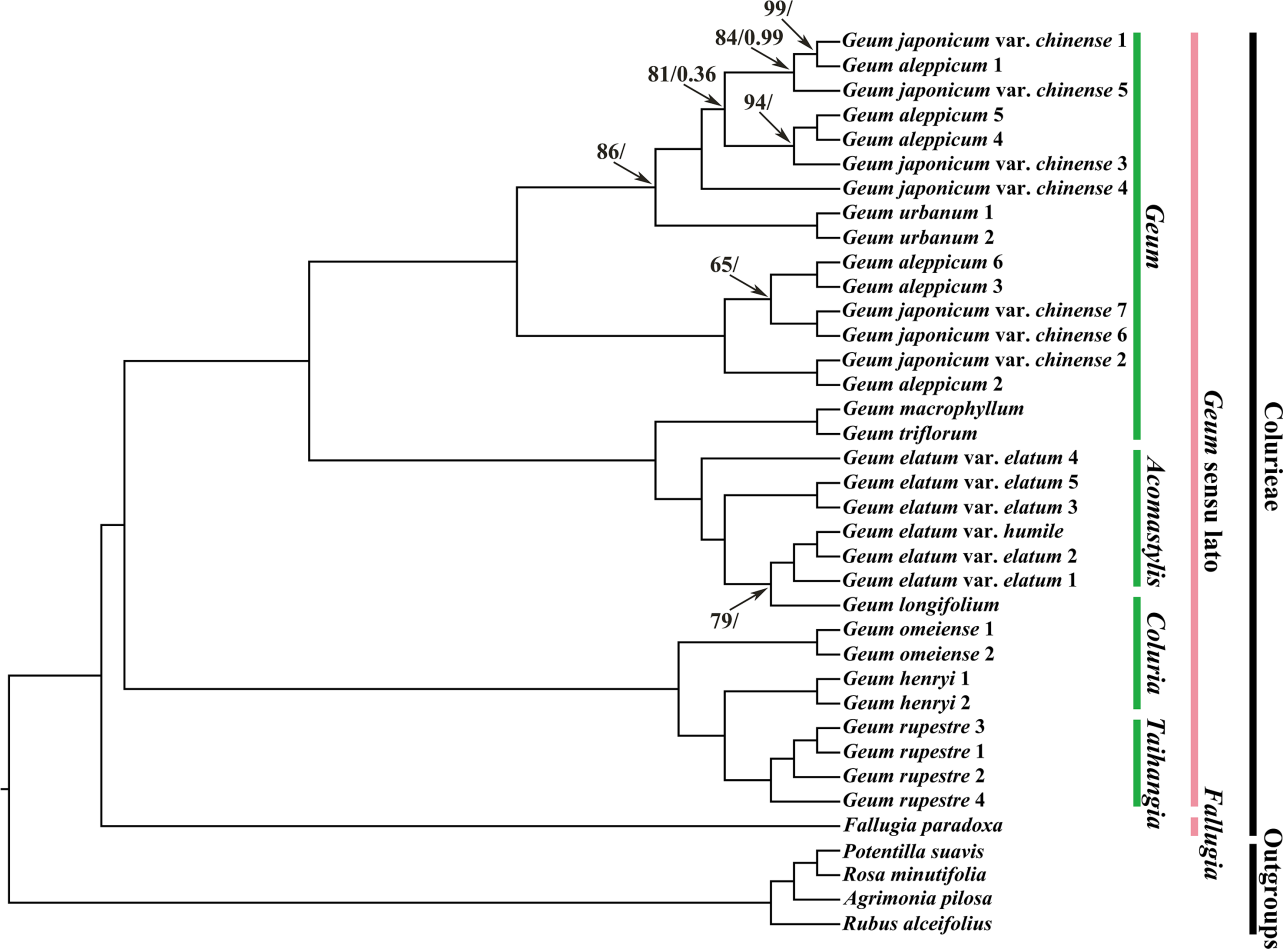
Phylogenetic trees of *Geum* and its related taxa based on 37 chloroplast genome sequences with the inverted repeat region IRa removed. *Agrimonia pilosa*, *Potentilla suavis*, *Rosa minutifolia*, and *Rubus alceifolius* were used to root the trees. Values along branches represent ML bootstrap percentages (only values < 100% are shown) and Bayesian posterior probabilities (only PP < 1.00 are shown), respectively.

Overall, compared with previous phylogenetic studies on *Geum* using cp molecular markers (Smedmark and Eriksson, 2002; Faghir et al., 2018; Protopopova et al., 2023), our study achieved higher phylogenetic resolution based on plastid genome data. Phylogenetic trees inferred using ML and BI analyses were consistent in topology (**Figure 4**; **Supplementary Figures S17, S18**). In all analyses, outgroups were robustly separated from the tribe Colurieae (ML BS = 100%, PP = 1.00). All sampled *Geum* species formed a well-supported clade (ML BS = 100%, PP = 1.00), confirming the monophyly of the current delimitation of *Geum*. Under the current sampling, two major clades were recovered within *Geum*. One comprised *G. rupestre*, *G. henryi*, and *G. omeiense*, with *G. omeiense* being sister to *G. henryi* and *G. rupestre.* The other consisted of two subclades: one including *G. longifolium*, *G. elatum* var. *elatum*, and *G. elatum* var. *humile*. *Geum triflorum*, and *G. macrophyllum*; the other consisting of *G. aleppicum*, *G. japonicum* var. *chinense*, and *Geum urbanum.* Multiple samples of *G. rupestre*, *G. henryi*, and *G. omeiense* clustered into separate branches, corroborating the monophyly of the three species and supporting their treatment as distinct species. A sample of *G. longifolium* was deeply nested within multiple samples of *G. elatum*, supporting the inclusion of *G. longifolium* in *G. elatum* as a conspecific taxon from a molecular phylogenetic perspective. *Geum longifolium* appears highly similar in morphology to *G. elatum*, especially in prominent features such as yellow flowers and basal, interrupted, pinnately compound leaves, but the most significant difference is that the former has wholly deciduous styles, whereas the latter has straight, nonplumose, persistent styles (Yü and Kuan, 1985; Yü and Li, 1985; Smedmark and Eriksson, 2002; Li et al., 2003). Future detailed morphological and phylogeographic studies based on a more comprehensive sampling strategy are necessary for any further possible taxonomic treatment of *G. longifolium*. Two samples of *G. urbanum*, which clustered together, were embedded within a large clade comprising different samples of *G. aleppicum* and *G. japonicum* var. *chinense* that were intermixed. The results showed that these three *Geum* taxa are nonmonophyletic. Morphologically, they are distinguished by differences such as petal size versus sepal size, shape of the capitulum of fruitlets, and the hair case of the receptacle (Juzepchuk, 1941; Yü and Kuan, 1985; Yü and Li, 1985; Li et al., 2003; Rohrer, 2014). Geographically, *G. urbanum* is native to Europe to Central Asia and Iran, NW. Africa; *G. aleppicum* is native to the temperate Northern Hemisphere to Mexico; and *G. japonicum* var. *chinense* is native to China, according to Plants of the World Online (http://powo.science.kew.org). The complicated evolutionary history and relationships among these three taxa still need to be clarified through expanded taxon sampling and the use of single‐copy nuclear genes in the future. *Geum elatum*, which was previously placed in *Acomastylis*, was nested within the species of *Geum*. Consistent with previous studies (Smedmark and Eriksson, 2002; Smedmark, 2006), our results supported the inclusion of *Acomastylis* species within *Geum*. *Geum longifolium*, *G. henryi*, and *G. omeiense*, which were once placed in *Coluria*, did not cluster together in our phylogenetic tree. *Geum longifolium* was closer to *G. elatum* than to *G. henryi* and *G. omeiense*, whereas *G. henryi* and *G. omeiense* showed a close affinity with *G. rupestre*, which was formerly treated as a member of the genus *Taihangia*.

Consistent with the studies of Smedmark and Eriksson (2002) and Faghir et al. (2018), our analyses indicated that *Coluria* is not a monophyletic group. The phylogenetic results presented here support treating *Coluria* as part of *Geum*. The genus *Taihangia* was nested within *Geum* species in Smedmark and Eriksson (2002). In conclusion, the results presented here supported Smedmark’s recircumscription of *Geum* in a broad sense and corroborate the inclusion of *Acomastylis*, *Coluria*, and *Taihangia* within *Geum*.

### 3.4 Adaptive evolution

In cases where the *p*-values of LRTs were below the threshold of 0.05 for the four compared models—M0 *vs*. M3, M1a *vs*. M2a, M7 *vs*. M8, and M8a *vs*. M8—the NEB analysis (Nielsen and Yang, 1998) was used to identify sites under positive selection with posterior probabilities ≥ 0.95 in model M3, while the BEB analysis (Yang et al., 2005) was used to identify sites under positive selection with posterior probabilities ≥ 0.95 in models M2a and M8, according to the PAML manual (Yang, 2007) (**Table 2**; **Supplementary Table S4**). The results showed that 48 genes had positive selection sites under model M3, none under model M2a, and 23 under model M8. The PAML manual (Yang, 2007) stated that the compared model M1a *vs*. M2a is more stringent than M7 *vs*. M8, which is corroborated by the results of our study. In addition, the PAML manual (Yang, 2007) suggested that the compared model M0 *vs*. M3 should be utilized to test for variable *ω* among sites, rather than as a test of positive selection. Therefore, we relied on the results from M7 *vs*. M8 and M8a *vs*. M8 to identify positively selected sites in *Geum* cp genomes. The BEB analysis based on the M8 model detected 90 positive selection sites across a total of 23 genes (**Table 2**). The number of the positive selection sites among these genes ranged from one to 39: *ycf1* with 39 sites; *ndhF* with 14 sites; three genes (*matk*, *rbcL*, and *accD*) with four sites; two genes (*rpl22* and *ndhD*) with three sites; three genes (*atpA*, *rpoC2*, and *psaA*) with two sites; and 13 genes (*atpF*, *rpoC1*, *rps4*, *cemA*, *psbJ*, *clpP*, *rps3*, *ycf2*, *ccsA*, *ndhI*, *ndhA*, *ndhH*, and *rps15*) with one site. These 23 PCGs with positively selected sites included three small subunit ribosomal genes (*rps3*, *rps4*, *rps15*); one large subunit ribosomal gene (*rpl22*); two DNA-dependent RNA polymerase (plastid-encoded bacterial-type RNA polymerase [PEP]) subunit genes (*rpoC1*, *rpoC2*); two ATP synthase subunit genes (*atpA*, *atpF*); two photosystem I (PSI) complex genes (*psaA*, *psaB*); a photosystem II (PSII) core complex gene (*psbJ*); five NAD(P)H dehydrogenase genes subunit genes (*ndhA*, *ndhD*, *ndhF*, *ndhI*, *ndhH*); the ribulose-1,5-bisphosphate carboxylase (Rubisco) large subunit gene (*rbcL*); the acetyl-CoA-carboxylase subunit gene (*accD*); the c-type cytochrome synthesis gene (*ccsA*); the cp membrane protein gene (*cemA*); the ATP-dependent Clp protease proteolytic subunit gene (*clpP*); the maturase gene (*matK*); as well as *ycf1* and *ycf2*.

**Table 2:** Positively selected sites (^*^p > 95%; ^**^p > 99%) identified in the chloroplast genomes of *Geum* in comparisons of M7 *vs*. M8 and M8a *vs*. M8 under Bayes empirical Bayes (BEB) analysis.

Gene	Positively selected sites	Pr(*w* > 1)	Number of sites
*matk*	472 V/604 K/643 K/830 P	0.969^*^/0.977^*^/0.974^*^/0.988^*^	4
*atpA*	1475 A/1628 A/	0.992^**^/0.969^*^	2
*atpF*	1645 T	0.985^*^	1
*rpoC2*	3303 Q/3319 D	0.961^*^/0.987^*^	2
*rpoC1*	4304 L	0.967^*^	1
*psaA*	7395 N/7452 S	0.975^*^/0.999^**^	2
*rps4*	8316 S	0.973^*^	1
*rbcL*	9578 H/9739 C/9941 C/9967 I	0.989^*^/0.993^**^/0.984^*^/0.997^**^	4
*accD*	10001 I/10034 D/10055 G/10107 M	0.952^*^/0.970^*^/0.980^*^/0.985^*^	4
*cemA*	10723 R	0.955^*^	1
*psbJ*	11266 A	0.989^*^	1
*clpP*	11989 Y	0.956^*^	1
*rps3*	14126 D	0.951^*^	1
*rpl22*	14398 L/14468 I/14473 G	0.954^*^/0.985^*^/0.998^**^	3
*ycf2*	15897 W	0.971^*^	1
*ndhF*	18372 V/18416 R/18418 S/18476 F/18493 Q/18517 I/18526 I/18544 S/18557 L/18574 L/18597 L/18664 C/18667 Y/18671–	0.980^*^/0.958^*^/0.983^*^/0.992^**^/0.967^*^/0.969^*^/0.969^*^/0.969^*^/0.966^*^/0.997^**^/0.964^*^/0.998^**^/1.000^**^/0.994^**^	14
*ccsA*	19009 F	0.979^*^	1
*ndhD*	19092 L/19500 K/19536 S	0.998^**^/0.987^*^/0.959^*^	3
*ndhI*	20069 F	0.978^*^	1
*ndhA*	20087 S	0.951^*^	1
*ndhH*	20743 K	0.963^*^	1
*rps15*	20924 L	0.961^*^	1
*ycf1*	21375 T/21392 K/21397 K/21420 A/21464 R/21477 I/21507 R/21534 S/21537 -/21562 L/21610 A/21614 P/21616 E/21649 I/21705 K/21706 K/21707 S/21709 L/21725 W/21730 K/21945 L/22009 V/22058 E/22106 I/22173 D/22180 K/22206 I/22242 Q/22288 R/22293 K/22322 M/22465 I/22469 D/22488 R/22544 L/22701 L/22742 N/22771 N/22878 N	0.995^**^/0.969^*^/0.965^*^/0.981^*^/0.968^*^/0.982^*^/0.968^*^/0.975^*^/0.986^*^/0.991^**^/0.970^*^/0.970^*^/0.983^*^/0.965^*^/0.999^**^/0.993^**^/0.993^**^/0.987^*^/0.997^**^/0.962^*^/0.999^**^/0.964^*^/0.968^*^/0.988^*^/0.981^*^/0.955^*^/0.991^**^/0.985^*^/0.984^*^/0.987^*^/0.975^*^/0.958^*^/0.984^*^/0.997^**^/0.981^*^/0.961^*^/0.967^*^/0.981^*^/0.980^*^	39

Amino acids refer to the sequence of *G. macrophyllum*.

*Geum* species occur in various habitats such as hillside grasslands, moist meadows, swamps, riverine scrub, rocky slopes, moist woods, rocky cliffs and ledges, alpine meadows, and arctic tundra, ranging from low to high altitudes (0–5,400 m) and often at high elevations (Juzepchuk, 1941; Smedmark and Eriksson, 2002; Li et al., 2003; Rohrer, 2014). The adaptive evolution of these 23 genes may contribute to the ability of *Geum* species to thrive in such diverse habitats. Genes such as *rps3*, *rps4*, *rps15*, and *rpl22* are ribosomal protein subunit genes that encode ribosomal proteins. The cp ribosomal proteins are important components of the protein synthesis machinery in all living cells, influencing plant growth and development and facilitating responses to stress conditions (Tiller and Bock, 2014; Zhang et al., 2016; Robles and Quesada, 2022). The plastid RNA polymerase subunits β’ and β”, encoded by genes *rpoC1* and *rpoC2*, respectively, are two of the four enzymatic subunits that constitute the catalytic core of the PEP (Zhelyazkova et al., 2012; Pfalz et al., 2015). The *atpA* and *atpF* genes encode two of the six ATP synthase subunits encoded by the plastome, and the cp ATP synthase generates the ATP needed for plant growth and photosynthesis (Wicke et al., 2011; Yamamoto et al., 2023). The *psaA* and *psaB* genes encode two major subunits of PSI, which bind to the iron–sulfur reaction center that mediates the majority of the electron transfer events (Nelson and Yocum, 2006; Wicke et al., 2011). The subunit PsbJ, encoded by the gene *psbJ*, is essential for the stable formation of PSII–light-harvesting complex (LHCII) supercomplexes, thereby enabling the higher-order organization of PSII complexes (Suorsa et al., 2004). The ndh genes, including *ndhA*, *ndhD*, *ndhF*, *ndhI*, and *ndhH*, encode subunits of the Ndh-1 complex, which plays a significant role in plant adaptation to environmental stress (Endo et al., 1999; Rumeau et al., 2007; Yamori et al., 2011). The gene *rbcL* encodes the large subunit of Rubisco (Wicke et al., 2011), and Rubisco mediates the fixation of inorganic carbon from CO_2_ into usable sugars during photosynthesis (Wilson and Hayer-Hartl, 2018; Whitney and Sharwood, 2021). The gene *cemA* encodes cp envelope membrane protein A, which is localized in the inner cp envelope membrane and mediates CO_2_ uptake (Sasaki et al., 1993; Katoh et al., 1996; Rolland et al., 1997). The gene *clpP* in the cp encodes one of the proteolytic subunits of the ATP-dependent Clp protease. Clp protease is involved in the degradation of polypeptides and is important for cp function, plant development, and stress acclimation (Clarke, 1999; Adam and Clarke, 2002; Kuroda and Maliga, 2003; Clarke et al., 2005). The gene *accD* encodes the beta-carboxyl transferase subunit of acetyl-CoA carboxylase (ACCase) (Wakasugi et al., 2001). ACCase in plastids is the regulatory enzyme of *de novo* fatty acid synthesis, which is crucial for leaf and seed development, storage metabolism, and cp division (Rawsthorne, 2002; Kode et al., 2005; Caroca et al., 2021). Cytochrome *c* biosynthesis protein, encoded by the *ccsA* gene, is essential for c-type cytochrome biosynthesis at the step of heme attachment (Xie and Merchant, 1996). Maturase K, encoded by the *matK* gene, is involved in the posttranscriptional processing of chloroplasts and is related to plant development and photosynthesis (Barthet and Hilu, 2007). The *ycf1* and *ycf2* genes are essential genes in the cp genomes of higher plants and encode products necessary for cell survival (Drescher et al., 2000). The origin of *Geum* was dated to the Miocene, 17 million years before the present (MYBP), with a 95% confidence interval from 10 to 26 MYBP (Smedmark et al., 2003; Smedmark, 2006). In conclusion, these 23 genes with sites under positive selection in the *Geum* cp genomes are associated with biological processes such as photosynthesis, biosynthesis, and self-replication, which may be key factors enabling the adaptation of *Geum* species to their habitats over evolutionary history.

## 4 Conclusion

In summary, comparative analyses showed that the 28 *Geum* cp genomes were conserved in structure, size, GC content, gene order, and gene content. Eleven regions (*3′-trnK-UUU-matK*, *psbZ-trnG-GCC*, *trnR-UCU-atpA*, *petA-psbJ*, *5′-trnK-UUU-rps16*, *rps16-trnQ-UUG*, *rpl32-trnL-UAG*, *ndhF-rpl32*, *trnS-GCU-trnG-UCC*, *ndhC-trnV-UAC*, and *petN-psbM*) may serve as candidate DNA molecular markers for future studies on population genetics and systematic evolution of *Geum* species. Our phylogenetic analyses provided new insights into the relationships among *Geum* species, supported Smedmark’s broad recircumscription of *Geum*, and corroborated the inclusion of *Acomastylis*, *Coluria*, and *Taihangia* within the genus. A total of 23 genes with positively selected sites were identified, suggesting that adaptive evolution of these genes may play important roles in the adaptation of *Geum* species to their habitats. Overall, this study offers valuable insights into cp genome characteristics, phylogeny, and adaptive evolution in *Geum*. Broader taxon sampling at a global scale and incorporation of single-copy nuclear genes will further clarify the phylogenetic relationships and evolutionary history of this group.

## Data Availability

The datasets presented in this study can be found in online repositories. The names of the repository/repositories and accession number(s) can be found in the article/**Supplementary Material**.
