# Supplementary material for: Comparative analyses of chloroplast genomes in Geum species: insights into genome characteristics, phylogenomic implications, and adaptive evolution
Source: Front Plant Sci. 2025 Dec 4;16:1713809. doi: 10.3389/fpls.2025.1713809 (PMC12809601; doi:10.3389/fpls.2025.1713809)
Supplement: Supplementary file 1 [file DataSheet1.zip › Supplementary Material/Captions for the Supplementary Material files.docx]

Supplementary Material

Comparative analyses of chloroplast genomes in *Geum* species: insights into genome characteristics, phylogenomic implications, and adaptive evolution

Wen-Tao Fu^1,2†^, Zhi-Ping Zhang^3†^, Jia-Jie Guo^1^, Jun Wen^4^, Qin-Qin Li^1,2*^

**†**These authors have contributed equally to this work

*** Correspondence:** Qin-Qin Li: liqq@imnu.edu.cn

**Supplementary Figure 1.** MAUVE alignment of complete chloroplast genomes of 28 accessions from nine *Geum* taxa, with *G. aleppicum* 1 as the reference.

**Supplementary Figure 2.** mVISTA identity plot based on Shuffle-LAGAN alignment of complete chloroplast genomes of 28 accessions from nine *Geum* taxa, with *G. aleppicum* 1 as the reference.

**Supplementary Figure 3.** Neighbor-joining (NJ) tree of *Geum* and its related taxa based on *3′-trnK-UUU-matK*. Values along branches represent the percentage bootstrap values of 1000 replicates.

**Supplementary Figure 4.** Neighbor-joining (NJ) tree of *Geum* and its related taxa based on *psbZ-trnG-GCC*. Values along branches represent the percentage bootstrap values of 1000 replicates.

**Supplementary Figure 5.** Neighbor-joining (NJ) tree of *Geum* and its related taxa based on *trnR-UCU-atpA*. Values along branches represent the percentage bootstrap values of 1000 replicates.

**Supplementary Figure 6.** Neighbor-joining (NJ) tree of *Geum* and its related taxa based on *petA-psbJ*. Values along branches represent the percentage bootstrap values of 1000 replicates.

**Supplementary Figure 7.** Neighbor-joining (NJ) tree of *Geum* and its related taxa based on *5′-trnK-UUU-rps16*. Values along branches represent the percentage bootstrap values of 1000 replicates.

**Supplementary Figure 8.** Neighbor-joining (NJ) tree of *Geum* and its related taxa based on *rps16-trnQ-UUG*. Values along branches represent the percentage bootstrap values of 1000 replicates.

**Supplementary Figure 9.** Neighbor-joining (NJ) tree of *Geum* and its related taxa based on *rpl32-trnL-UAG*. Values along branches represent the percentage bootstrap values of 1000 replicates.

**Supplementary Figure 10.** Neighbor-joining (NJ) tree of *Geum* and its related taxa based on *ndhF-rpl32*. Values along branches represent the percentage bootstrap values of 1000 replicates.

**Supplementary Figure 11.** Neighbor-joining (NJ) tree of *Geum* and its related taxa based on *trnS-GCU-trnG-UCC*. Values along branches represent the percentage bootstrap values of 1000 replicates.

**Supplementary Figure 12.** Neighbor-joining (NJ) tree of *Geum* and its related taxa based on *ndhC-trnV-UAC*. Values along branches represent the percentage bootstrap values of 1000 replicates.

**Supplementary Figure 13.** Neighbor-joining (NJ) tree of *Geum* and its related taxa based on *petN-psbM*. Values along branches represent the percentage bootstrap values of 1000 replicates.

**Supplementary Figure 14.** Neighbor-joining (NJ) tree of *Geum* and its related taxa based on *trnH-GUG-psbA*. Values along branches represent the percentage bootstrap values of 1000 replicates.

**Supplementary Figure 15.** Neighbor-joining (NJ) tree of *Geum* and its related taxa based on *rbcL*. Values along branches represent the percentage bootstrap values of 1000 replicates.

**Supplementary Figure 16.** Neighbor-joining (NJ) tree of *Geum* and its related taxa based on *matK*. Values along branches represent the percentage bootstrap values of 1000 replicates.

**Supplementary Figure 17.** Maximum likelihood (ML) tree of *Geum* and its related taxa based on 37 chloroplast genome sequences with the inverted repeat region IRa removed. *Agrimonia pilosa*, *Potentilla suavis*, *Rosa minutifolia*, and *Rubus alceifolius* were used to root the tree. Values along branches represent ML bootstrap percentages (only values < 100 % are shown).

**Supplementary Figure 18.** Bayesian inference (BI) tree of *Geum* and its related taxa based on 37 chloroplast genome sequences with the inverted repeat region IRa removed. *Agrimonia pilosa*, *Potentilla suavis*, *Rosa minutifolia*, and *Rubus alceifolius* were used to root the tree. Values along branches represent Bayesian posterior probabilities (only PP < 1.00 are shown).

**Supplementary Table 1**. The GenBank accession numbers of all the 37 chloroplast genome sequences used for phylogenetic analyses.

**Supplementary Table 2**. Genes contained in the *Geum* chloroplast genomes.

**Supplementary Table 3**. The nucleotide diversity (Pi) values of 264 homologous loci in 28 *Geum* chloroplast genomes.

**Supplementary Table 4.** Positively selected sites (*: P>95%; **: P>99%) identiﬁed in the chloroplast genomes of *Geum* in comparison of M0 vs. M3 under Naïve empirical Bayes (NEB) analysis. Amino acids refer to sequence of *G. macrophyllum*.
