# Supplementary material for: Comparative analyses of chloroplast genomes in Geum species: insights into genome characteristics, phylogenomic implications, and adaptive evolution
Source: Front Plant Sci. 2025 Dec 4;16:1713809. doi: 10.3389/fpls.2025.1713809 (PMC12809601; doi:10.3389/fpls.2025.1713809)
Supplement: Supplementary file 1 [file DataSheet1.zip › Supplementary Material/Table S2.docx]

Table S2. Genes contained in the *Geum* chloroplast genomes.

| Category of genes | Group of genes | Name of genes |
| --- | --- | --- |
| Self-replication | Ribosomal RNAs | *rrn4.5*^a^*, rrn5*^a^*, rrn16*^a^, *rrn23*^a^ |
|  | Transfer RNAs | *trnA-UGC*^a*^, *trnC-GCA*, *trnD-GUC*, *trnE-UUC*, *trnF-GAA*, *trnfM-CAU*, *trnG-GCC*, *trnG-UCC*^*^, *trnH-GUG*, *trnI-CAU*^a^, *trnI-GAU*^a*^, *trnK-UUU*^*^, *trnL-CAA*^a^, *trnL-UAA*^*^, *trnL-UAG*, *trnM-CAU*, *trnN-GUU*^a^, *trnP-UGG*, *trnQ-UUG*, *trnR-ACG*^a^, *trnR-UCU*, *trnS-GCU*, *trnS-GGA*, *trnS-UGA*, *trnT-GGU*, *trnT-UGU*, *trnV-GAC*^a^, *trnV-UAC*^*^, *trnW-CCA*, *trnY-GUA* |
|  | Small subunit of ribosome | *rps2*, *rps3*, *rps4*, *rps7*^a^, *rps8*, *rps11*, *rps12*^ab**^, *rps14*, *rps15*, *rps16*^*^, *rps18*, *rps19* |
|  | Large subunit of ribosome | *rpl2*^a*^, *rpl14*, *rpl16*^*^, *rpl20*, *rpl22*, *rpl23*^a^, *rpl32*, *rpl33*, *rpl36* |
|  | DNA dependent RNA polymerase | *rpoA*, *rpoB*, *rpoC1*^*^, *rpoC2* |
| Photosynthesis | Subunits of ATP synthase | *atpA*, *atpB*, *atpE*, *atpF*, *atpH*, *atpI* |
|  | Subunits of photosystem I | *psaA*, *psaB*, *psaC*, *psaI*, *psaJ*, *ycf3*^**^, *ycf4* |
|  | Subunits of photosystem II | *psbA*, *psbB*, *psbC*, *psbD*, *psbE*, *psbF*, *psbH*, *psbI*, *psbJ*, *psbK*, *psbL*, *psbM*, *psbN*, *psbT*, *psbZ* |
|  | Subunits of cytochrome b/f complex | *petA*, *petB*^*^, *petD*^*^, *petG*, *petL*, *petN* |
|  | Subunits of NADH-dehydrogenase | *ndhA*^*^, *ndhB*^a*^, *ndhC*, *ndhD*, *ndhE*, *ndhF*, *ndhG*, *ndhH*, *ndhI*, *ndhJ*, *ndhK* |
|  | Subunit of Rubisco | *rbcL* |
| Other genes | Subunit of Acetyl-CoA-carboxylase | *accD* |
|  | C-type cytochrome synthesis | *ccsA* |
|  | Envelop membrane protein | *cemA* |
|  | Protease | *clpP*^**^ |
|  | Maturase | *matK* |
| Unkown function | Conserved open reading frame | *ycf1*, *ycf2*^a^ |

^a^ Two gene copies in IRs; ^b^ gene divided into two independent transcription units; ^*^ genes containing one intron; ^**^ genes containing two introns.
