# Supplementary material for: Comparative analyses of chloroplast genomes in Geum species: insights into genome characteristics, phylogenomic implications, and adaptive evolution
Source: Front Plant Sci. 2025 Dec 4;16:1713809. doi: 10.3389/fpls.2025.1713809 (PMC12809601; doi:10.3389/fpls.2025.1713809)
Supplement: Supplementary file 1 [file DataSheet1.zip › Supplementary Material/Table S4.docx]

Table S4. Positively selected sites (*: P>95%; **: P>99%) identiﬁed in the chloroplast genomes of *Geum* in comparison of M0 vs. M3 under Naïve empirical Bayes (NEB) analysis. Amino acids refer to sequence of *G. macrophyllum.*

| Gene | Positively selected sites | Pr(w>1) | Number of sites |
| --- | --- | --- | --- |
| *matk* | 367 W/392 G/394 I/422 H/434 K/435 K/436 L/  437 G/446 I/472 V/475 S/548 P/549 K/550 D/  560 L/587 R/599 R/604 K/618 T/640V/643 K/  657 N/678 G/684 V/733 K/829 F/830 P/856 I | 0.982*/0.999**/0.950*/0.958*/0.999**/  0.989*/0.976*/0.981*/0.998**/1.000**/  0.962*/0.971*/0.960*/0.964*/0.971*/0.980*/0.999**/1.000**/0.982*/0.962*/1.000**/  0.952*/0.984*/0.954*/0.958*/0.958*/1.000**/0.970* | 28 |
| *rps16* | 930 F | 0.988* | 1 |
| *psbK* | 987 I | 0.950* | 1 |
| *atpA* | 1475 A/1543 M | 1.000**/0.958* | 2 |
| *atpF* | 1628 A/1645 T | 0.999**/1.000** | 2 |
| *rpoC2* | 2309 N/2383 L/2526 I/2587 H/2695 E/2733 F/  2785 C/2790 G/2812 D/2813 I/2818 L/2825 H/  2841 C/2843 P/2852 I/2895 S/2953 I/3021 K/  3048 T/3128 S/3153 H/3161 V/3215 R/3256 G/  3277 K/3303 Q/3306 V/3317 L/3319 D/  3363 N/3374 T/3393 P/3553 S/3656 K/  3658 G/3673 E/3676 D | 0.966*/0.978*/0.974*/0.964*/0.958*/0.951*/0.983*/0.980*/0.959*/0.960*/0.969*/  0.979*/0.964*/0.964*/0.956*/0.984*/0.980*/0.957*/0.963*/0.950*/0.980*/0.966*/  0.960*/0.965*/0.999**/0.999**/0.999**/  0.971*/1.000**/0.967*/0.964*/0.976*/  0.974*/0.967*/0.953*/0.959*/0.963* | 37 |
| *rpoC1* | 4180 D/4266 S/4277 D/4304 L/4305 G | 0.965*/0.971*/0.980*/0.999**/0.950* | 5 |
| *rpoB* | 4396 A/4403 L/4424 Y/4975 I/4984 V/5068 G/  5170 K/5264 F | 0.979*/0.951*/0.961*/0.979*/0.950*/0.969*/0.965*/0.964* | 8 |
| *psbC* | 6154 A/6301 A | 0.999**/0.998** | 2 |
| *psaB* | 6588 H/6715 S/7010 V | 0.973*/0.976*/0.952* | 3 |
| *psaA* | 7391 S/7395 N/7452 S/7606 I/7843 A | 0.965*/1.000**/1.000**/0.998**/0.965* | 5 |
| *rps4* | 8316 S/8328 Q | 1.000**/0.988* | 2 |
| *ndhJ* | 8455 R | 0.975* | 1 |
| *ndhK* | 8535 K/8695 C | 0.970*/0.964* | 2 |
| *ndhC* | 8769 F/8772 L | 0.970*/0.977* | 2 |
| *atpE* | 8956 P | 0.987* | 1 |
| *atpB* | 9009 G/9013 A/9228 P/9383 D/9491 E | 0.958*/0.983*/0.984*/0.999**/0.964* | 5 |
| *rbcL* | 9578 H/9583 P/9593 V/9634 P/9718 F/9739 C/  9741 E/9747 I/9771 S/9820 A/9846 I/9941 C/  9967 I | 1.000**/0.963*/0.965*/0.964*/0.998**/  1.000**/0.998**/0.957*/0.979*/0.960*/  0.952*/1.000**/1.000** | 13 |
| *accD* | 9989 N/9993 A/9994 C/10001 I/10009 L/  10034 D/10055 G/10065 K/10073 R/10088 T/  10095 P/10102 R/10104 D/10105 T/10107 M/  10133 C/10137 Y/10159 T/10162 S/10175 I/  10178 G/10224 C/10236 L/10327 S/10434 P/  10440 L | 0.954*/0.982*/0.998**/0.999**/0.966*/  1.000**/1.000**/0.963*/0.977*/0.953*/  0.967*/0.984*/0.952*/0.968*/1.000**/  0.963*/0.961*/0.999**/0.998**/0.958*/  0.961*/0.999**/0.960*/0.960*/0.971*/  0.999** | 26 |
| *ycf4* | 10545 L | 0.963* | 1 |
| *cemA* | 10700 F/10723 R/10736 S/10767 L/10775 Q/  10779 M/10889 C | 0.979*/0.999**/0.998**/0.979*/0.972*/  0.956*/0.976* | 7 |
| *petA* | 10947 I/11105 A | 0.967*/0.956* | 2 |
| *psbJ* | 11266 A | 1.000** | 1 |
| *psbE* | 11438 S | 0.964* | 1 |
| *rpl33* | 11581 R/11589 L/11615 Y | 0.979*/0.981*/0.963* | 3 |
| *rps18* | 11701 P/11714 N /11718 A | 0.983*/0.978*/0.987* | 3 |
| *rpl20* | 11801 D/11810 K | 0.967*/0.958* | 2 |
| *clpP* | 11989 Y/12008 I/12039 S/12043 Y/12045 G/  12061 V/12102 G/12144 L | 0.999**/0.993**/0.964*/0.983*/0.961*/  0.954*/0.966*/0.969* | 8 |
| *psbB* | 12188 S/12288 A/12661 A/12906 V/13034 L | 0.963*/0.964*/0.965*/0.954*/0.972* | 5 |
| *petD* | 13183 V | 0.969* | 1 |
| *rpoA* | 13272 F/13335 I/13468 G/13473 A/13486 P/  13517 V/13534 N/13535 L/13536 E | 0.966*/0.998**/0.998**/0.972*/0.984*/  0.970*/0.952*/0.975*/0.982* | 9 |
| *rps11* | 13561 A/13616 G | 0.980*/0.968* | 2 |
| *rps8* | 13786 P | 0.972* | 1 |
| *rpl14* | 13857 Y/13868 K/13925 I/13999 R | 0.957*/0.998**/0.953*/0.967* | 4 |
| *rps3* | 14126 D/14160 M/14184 I/14222 A/14227 A | 0.999**/0.974*/0.955*/0.967*/0.962* | 5 |
| *rpl22* | 14339 -/14340 M/14347 P/14361 R/14398 L/  14414 F/14443 S/14468 I/14469 D/14470 K/  14471 R/14472 K/14473 G/14474 Y/14477 L/  14480 K/14486 L | 0.976*/0.986*/0.978*/0.964*/0.999**/  0.983*/0.954*/1.000**/0.999**/0.962*/  0.978*/0.966*/1.000**/0.993**/0.991**/  0.979*/0.965* | 17 |
| *ycf2* | 14999 F/15556 S/15897 W/16066 Y/16147 L/  16244 M/16246 G/16248 D/16319 L/16893 R/  16959 -/17054 W | 0.971*/0.975*/1.000**/0.954*/0.967*/  0.994**/0.970*/0.971*/0.983*/0.979*/  0.990*/0.982* | 12 |
| *ndhF* | 17962 V/17965 V/17969 L/17999 Y/18001 L/  18043 F/18099 F/18198 V/18205 I/18365 I/  18372 V/18382 S/18416 R/18417 T/18418 S/  18422 R/18426 T/18434 A/18436 K/18442 P/  18443 H/18476 F/18477 V/18490 N/18493 Q/  18496 I/18497 N/18517 I/18526 I/18535 I/  18538 S/18544 S/18557 L/18560 F/18566 K/  18571 I/18574 L/18579 L/18594 A/18597 L/  18603 G/18606 K/18632 L/18660 L/18664 C/  18665 Y/18667 Y/18671 - | 0.998**/0.998**/0.960*/0.988*/0.998**/  0.979*/0.951*/0.954*/0.969*/0.954*/1.000**/0.975*/0.999**/0.967*/1.000**/0.995**/0.998**/0.966*/0.977*/0.959*/0.979*/  1.000**/0.999**/0.983*/0.999**/0.999**/  0.998**/0.999**/1.000**/0.952*/0.981*/  1.000**/0.999**/0.952*/0.975*/0.953*/  1.000**/0.965*/0.952*/0.999**/0.958*/  0.964*/0.975*/0.973*/1.000**/0.999**/  1.000**/1.000** | 48 |
| *rpl32* | 18692 I/18695 R/18715 N | 0.952*/0.975*/0.977* | 3 |
| *ccsA* | 18735 H/18784 I/18821 N/18895 S/18909 M/  18911 S/18923 K/18928 Q/18929 N/18933 L/  18935 Y/18999 V/19007 K/19009 F/19048 P | 0.963*/0.998**/0.998**/0.980*/0.965*/  0.982*/0.962*/0.973*/0.973*/0.950*/0.965*/0.950*/0.957*/1.000**/0.976* | 15 |
| *ndhD* | 19060 V/19066 S/19082 I/19083 K/19088 C/  19092 L/19102 A/19151 T/19178 L/19226 I/  19466 I/19500 K/19533 F/19536 S/19551 S | 0.954*/0.983*/0.953*/0.975*/0.999**/  1.000**/0.964*/0.953*/0.970*/0.952*/  0.958*/1.000**/0.976*/0.999**/0.999** | 15 |
| *ndhE* | 19636 F | 0.980* | 1 |
| *ndhG* | 19757 S/19820 Y/19833 V/19836 S/19847 I | 0.999**/0.963*/0.950*/0.970*/0.952* | 5 |
| *ndhI* | 19926 V/19935 G/20021 V/20069 F | 0.961*/0.955*/0.950*/1.000** | 4 |
| *ndhA* | 20087 S/20091 A/20097 K/20169 L/20192 V/  20285 G/20300 L/20383 V/20393 A | 0.999**/0.951*/0.999**/0.959*/0.962*/  0.981*/0.981*/0.967*/0.957* | 9 |
| *ndhH* | 20456 V/20458 S/20585 I/20674 Q/20713 I/  20743 K/20745 I/20783 I | 0.960*/0.951*/0.966*/0.972*/0.999**/  0.999**/0.975*/0.952* | 8 |
| *rps15* | 20909 R/20924 L | 0.963*/0.999** | 2 |
| *ycf1* | 21252 N/21261 G/21281 D/21288 D/21304 K/  21319 -/21341 T/21373 Q/21375 T/21377 S/  21378 P/21386 Y/21392 K/21395 N/21397 K/  21403 N/21405 L/21410 T/21412 D/21420 A/  21425 V/21440 I/21464 R/21477 I/21487 C/  21507 R/21512 R/21514 T/21515 F/21516 V/  21520 N/21528 R/21530 Y/21534 S/21536 H/  21537 -/21538 -/21541 N/21545 I/21546 S/  21560 K/21562 L/21563 K/21568 S/21570 A/  21573 P/21577 P/21578 I/21589 Q/21608 A/  21610 A/21611 E/21613 H/21614 P/21616 E/  21636 Q/21642 -/21648 E/21649 I/21705 K/  21706 K/21707 S/21709 L/21725 W/21727 W/  21730 K/21733 Q/21749 N/21798 I/21809 V/  21827 K/21852 I/21895 V/21912 S/21918 F/  21925 L/21928 L/21932 Y/21937 K/21939 F/  21943 I/21945 L/21947 L/21956 R/21960 S/  21962 L/21972 S/21976 L/21983 S/21985 R/  21991 T/21993 N/22007 H/22009 V/22018 I/  22044 G/22058 E/22059 G/22061 L/22068 S/  22078 E/22083 S/22084 K/22088 G/22096 G/  22106 I/22107 I/22132 A/22134 E/22145 P/  22154 S/22157 T/22168 I/22171 V/22173 D/  22177 I/22178 N/22180 K/22182 S/22184 T/  22187 D/22204 K/22206 I/22208 L/22215 P/  22217 F/22224 P/22233 C/22240 L/22242 Q/  22246 R/22255 M/22259 Q/22282 S/22288 R/  22293 K/22296 L/22309 T/22320 K/22322 M/  22324 M/22344 Q/22356 N/22363 L/22365 L/  22380 Q/22382 R/22384 S/22390 D/22406 R/  22412 L/22442 Q/22451 A/22457 K/22465 I/  22466 K/22469 D/22474 T/22486 Q/22488 R/  22502 H/22509 R/22518 L/22544 L/22553 V/  22558 -/22580 G/22588 A/22607 M/22610 D/  22611 I/22635 H/22701 L/22733 P/22742 N/  22745 N/22771 N/22796 N/22802 T/22810 T/  22820 P/22827 V/22828 D/22829 K/22865 S/  22878 N | 0.980*/0.965*/0.974*/0.987*/0.961*/0.992**/0.961*/0.972*/1.000**/0.972*/0.976*/  0.999**/1.000**/0.957*/0.999**/0.983*/  0.961*/0.957*/0.968*/1.000**/0.952*/  0.951*/0.999**/1.000**/0.977*/0.999**/  0.971*/0.964*/0.981*/0.964*/0.998**/  0.961*/0.964*/1.000**/0.956*/1.000**/  0.952*/0.967*/0.951*/0.999**/0.958*/  1.000**/0.956*/0.966*/0.999**/0.984*/  0.979*/0.998**/0.999**/0.969*/1.000**/  0.970*/0.998**/1.000**/1.000**/0.979*/  0.968*/0.958*/0.999**/1.000**/1.000**/  1.000**/1.000**/1.000**/0.983*/0.999**/  0.964*/0.973*/0.950*/0.979*/0.954*/0.958*/0.950*/0.975*/0.951*/0.955*/0.979*/  0.983*/0.961*/0.998**/0.959*/1.000**/  0.976*/0.985*/0.981*/0.985*/0.974*/0.973*/0.969*/0.964*/0.961*/0.950*/0.959*/  0.999**/0.998**/0.969*/1.000**/0.968*/  0.994**/0.957*/0.953*/0.974*/0.968*/  0.983*/0.998**/1.000**/0.998**/0.998**/  0.961*/0.983*/0.998**/0.965*/0.960*/  0.962*/1.000**/0.950*/0.952*/0.999**/  0.973*/0.955*/0.975*/0.999**/1.000**/  0.974*/0.966*/0.951*/0.965*/0.964*/0.986*/1.000**/0.963*/0.962*/0.969*/0.970*/  1.000**/1.000**/0.994**/0.976*/0.998**/  1.000**/0.958*/0.986*/0.973*/0.993**/  0.971*/0.999**/0.971*/0.978*/0.956*/  0.992**/0.981*/0.977*/0.958*/0.969*/  0.999**/0.956*/1.000**/0.978*/0.973*/  1.000**/0.968*/0.967*/0.959*/1.000**/  0.958*/0.978*/0.992**/0.966*/0.973*/  0.975*/0.954*/0.960*/0.999**/0.972*/  0.999**/0.998**/1.000**/0.998**/0.967*/  0.963*/0.996**/0.968*/0.999**/0.960*/  0.998**/1.000** | 186 |
|  |  |  |  |
