# Supplementary figures and images for: Comparative analyses of chloroplast genomes in Geum species: insights into genome characteristics, phylogenomic implications, and adaptive evolution
Source: Front Plant Sci. 2025 Dec 4;16:1713809. doi: 10.3389/fpls.2025.1713809 (PMC12809601; doi:10.3389/fpls.2025.1713809)

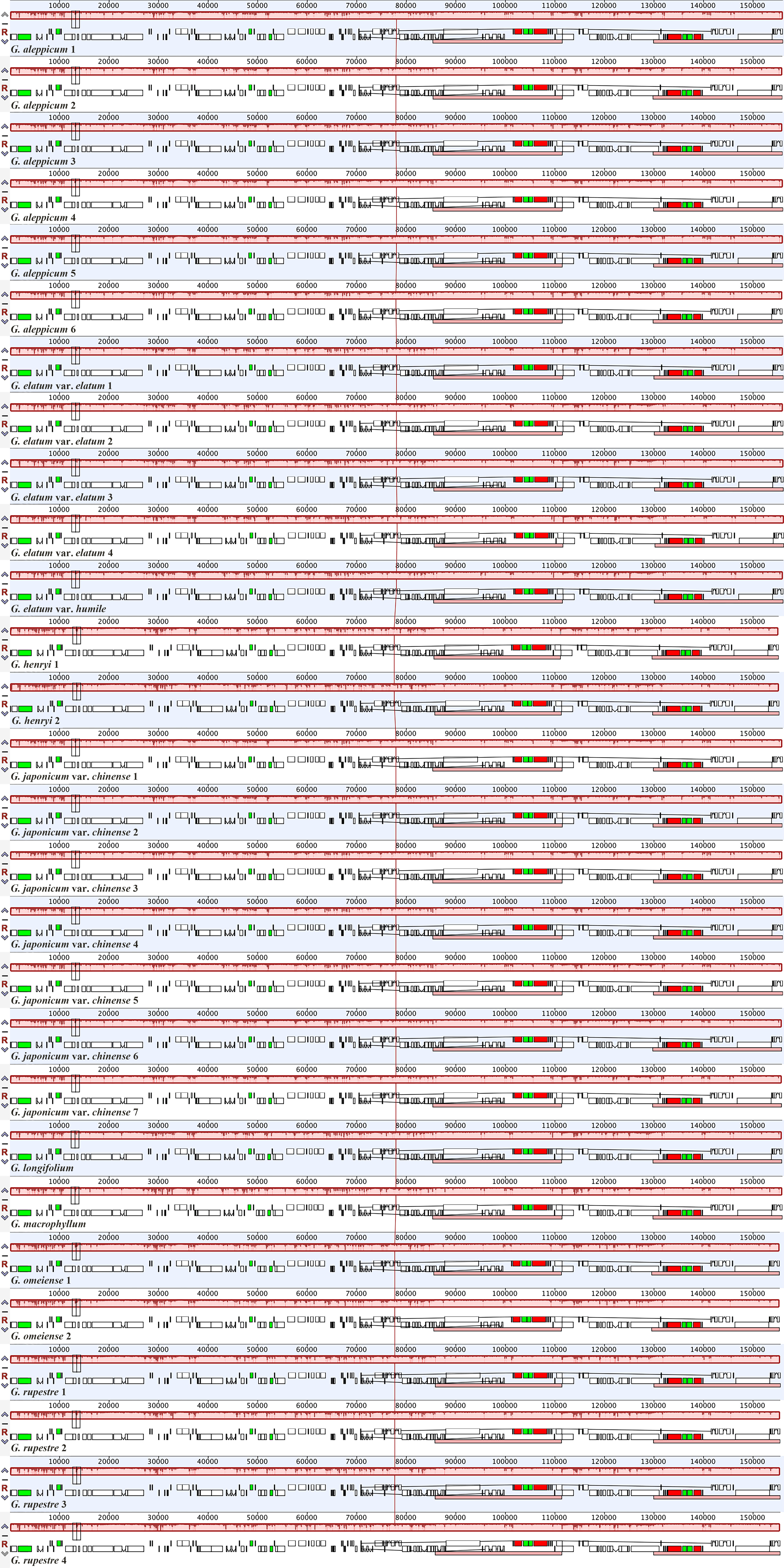

Supplement: Supplementary file 1 [file DataSheet1.zip › Supplementary Material/Figure S1.jpg]

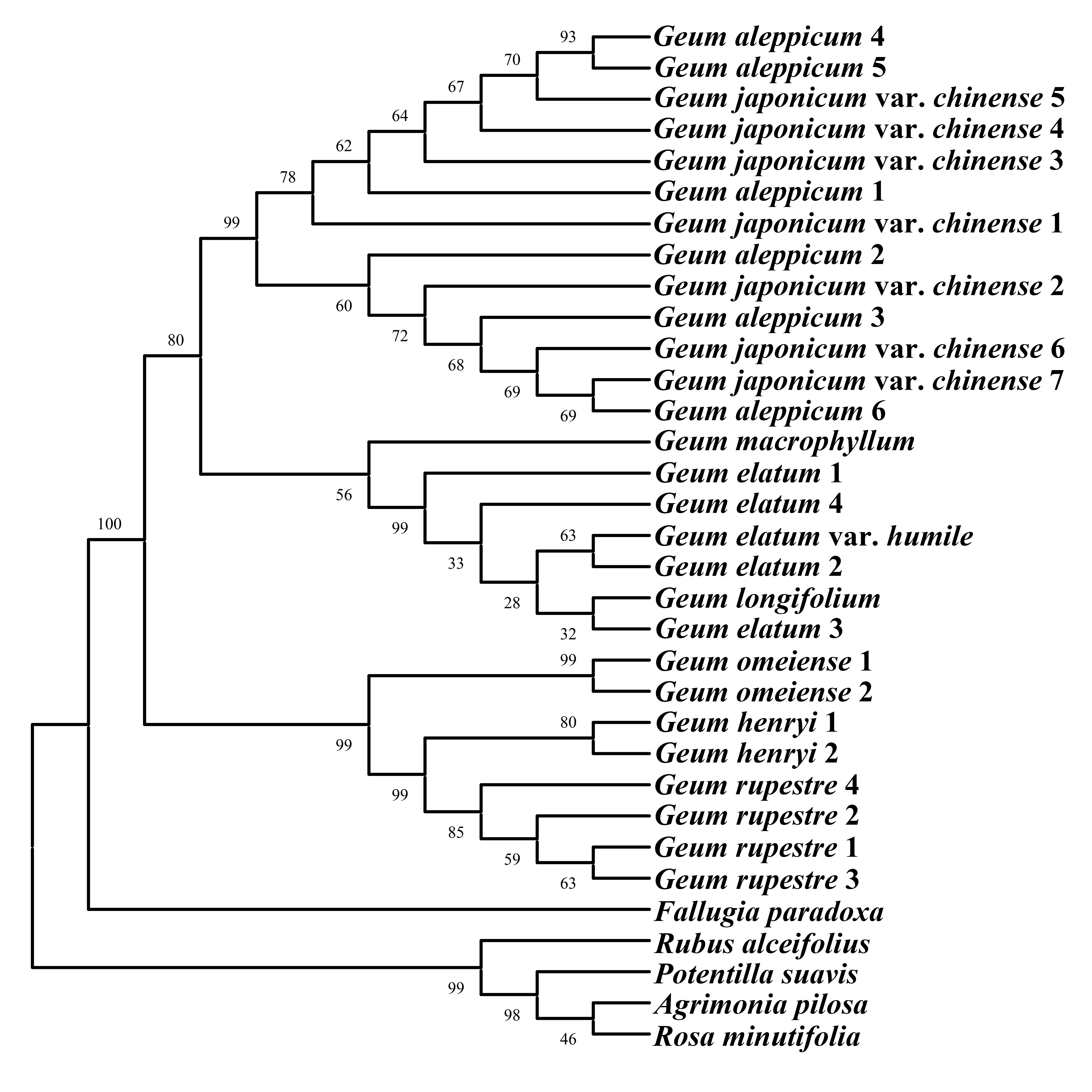

Supplement: Supplementary file 1 [file DataSheet1.zip › Supplementary Material/Figure S10.jpg]

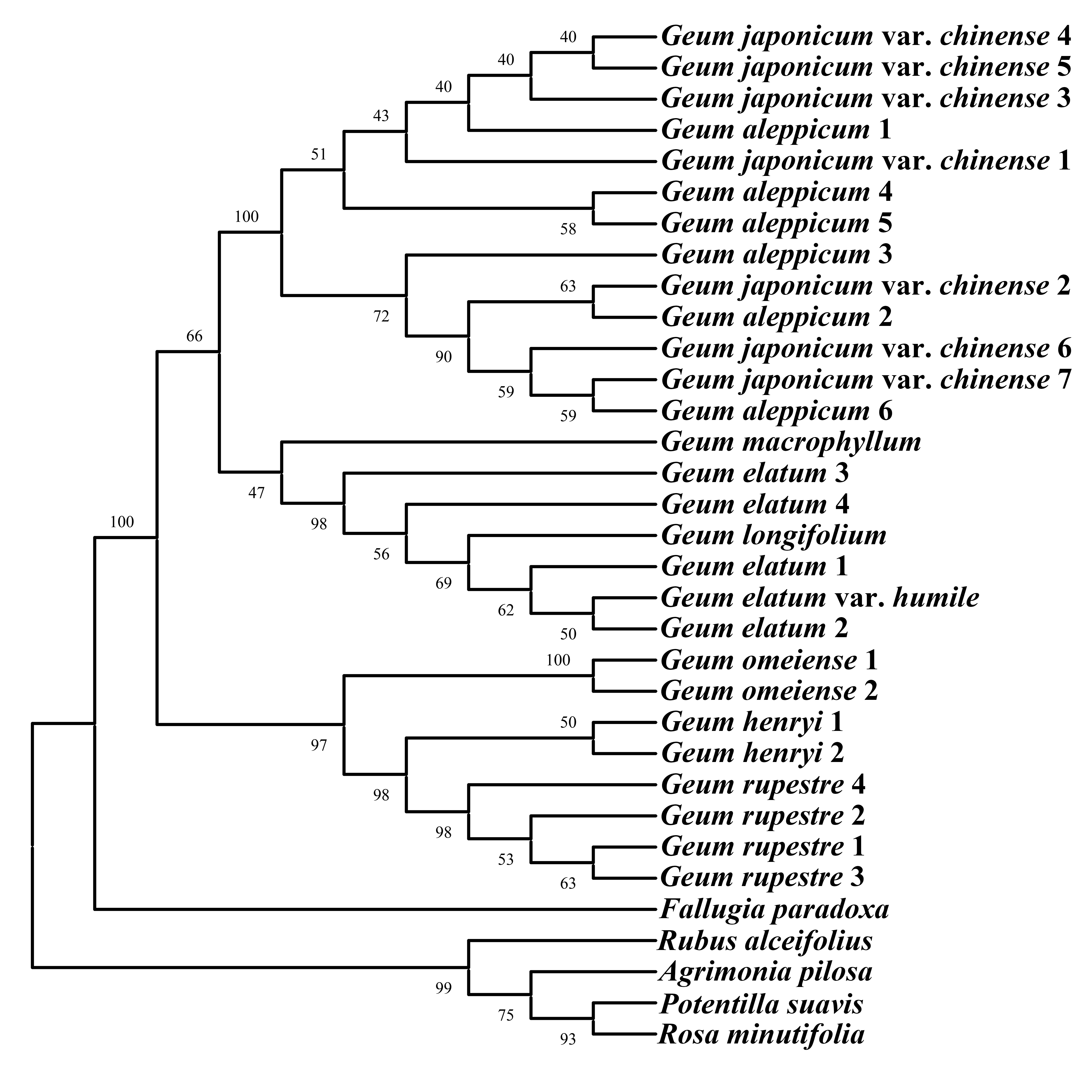

Supplement: Supplementary file 1 [file DataSheet1.zip › Supplementary Material/Figure S11.jpg]

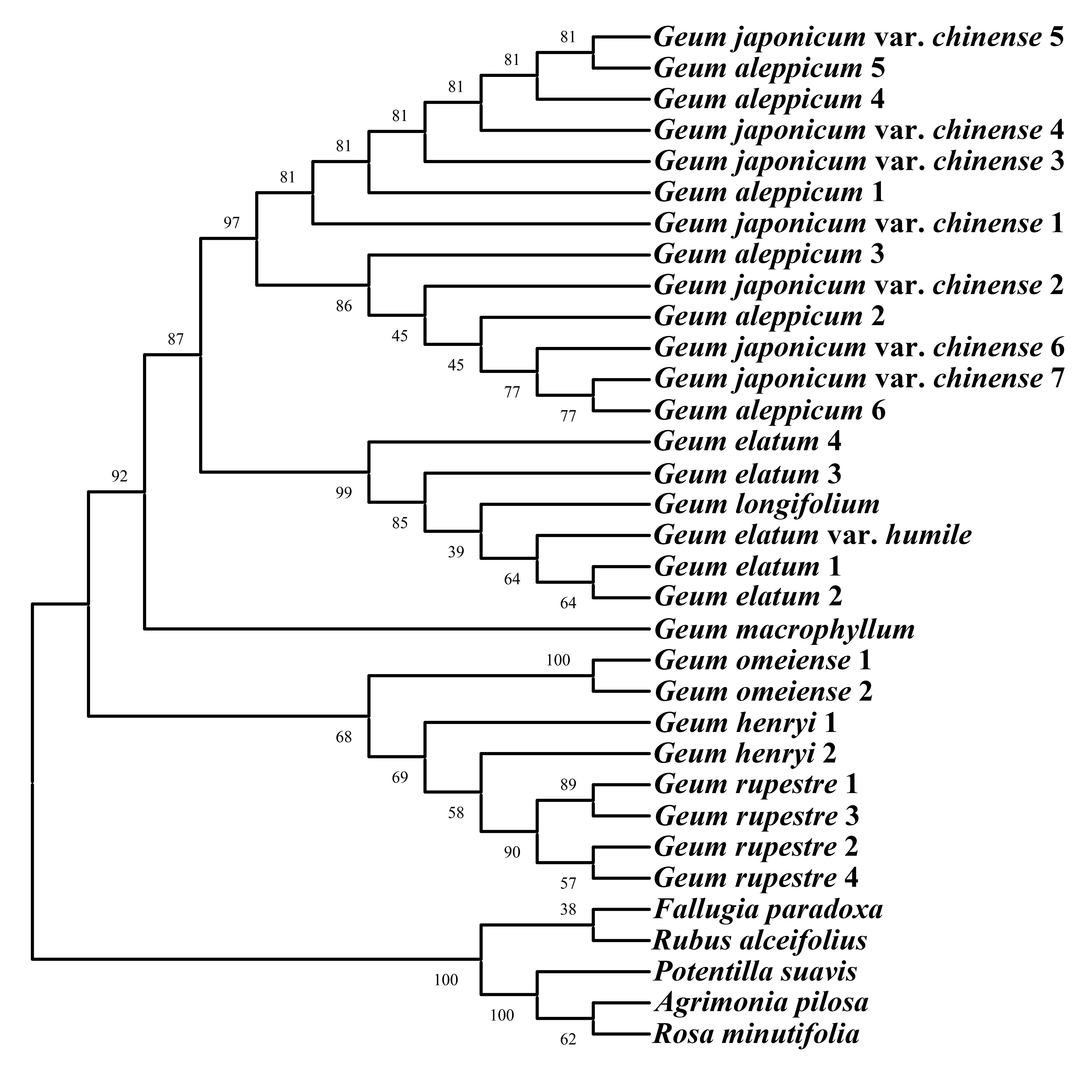

Supplement: Supplementary file 1 [file DataSheet1.zip › Supplementary Material/Figure S12.jpg]

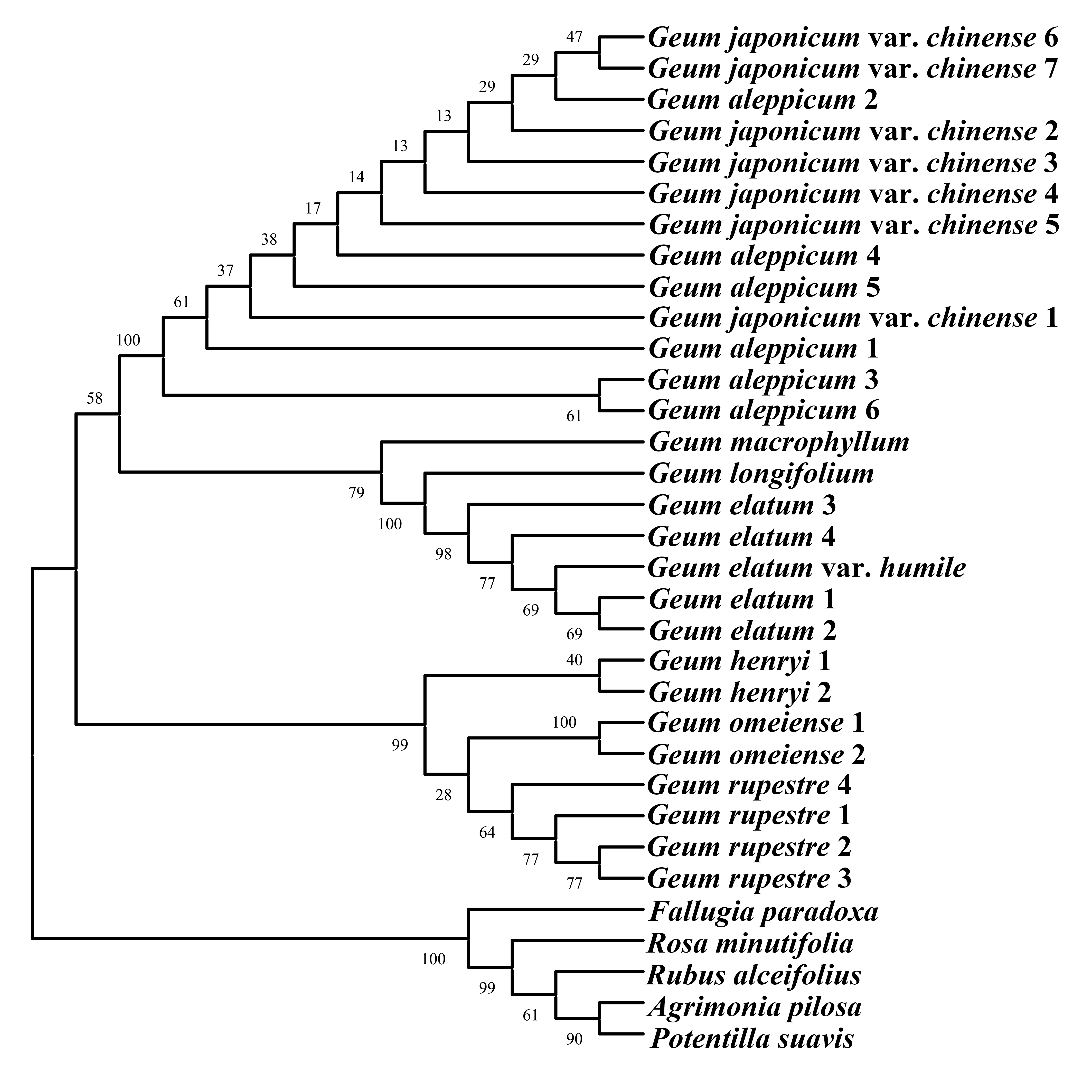

Supplement: Supplementary file 1 [file DataSheet1.zip › Supplementary Material/Figure S13.jpg]

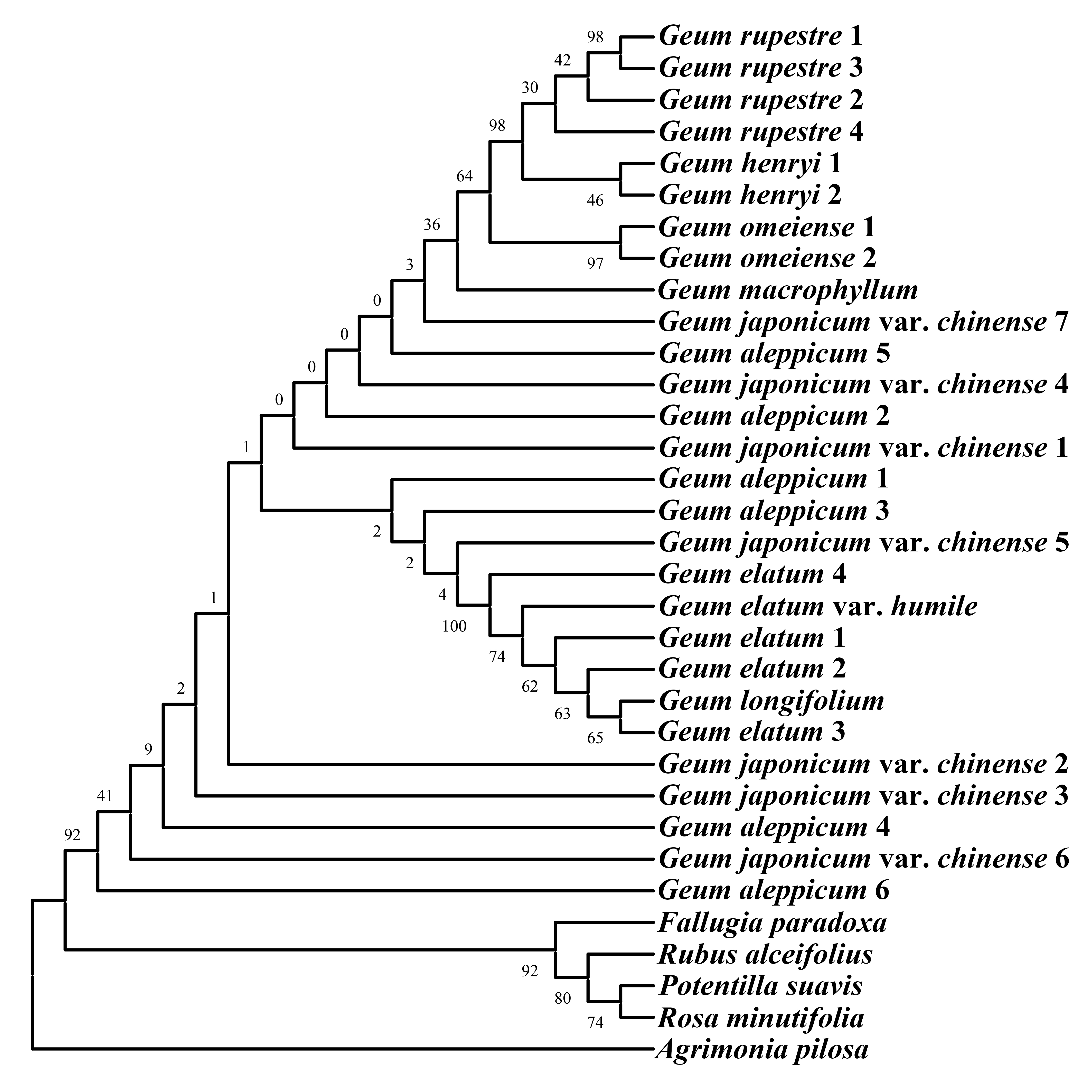

Supplement: Supplementary file 1 [file DataSheet1.zip › Supplementary Material/Figure S14.jpg]

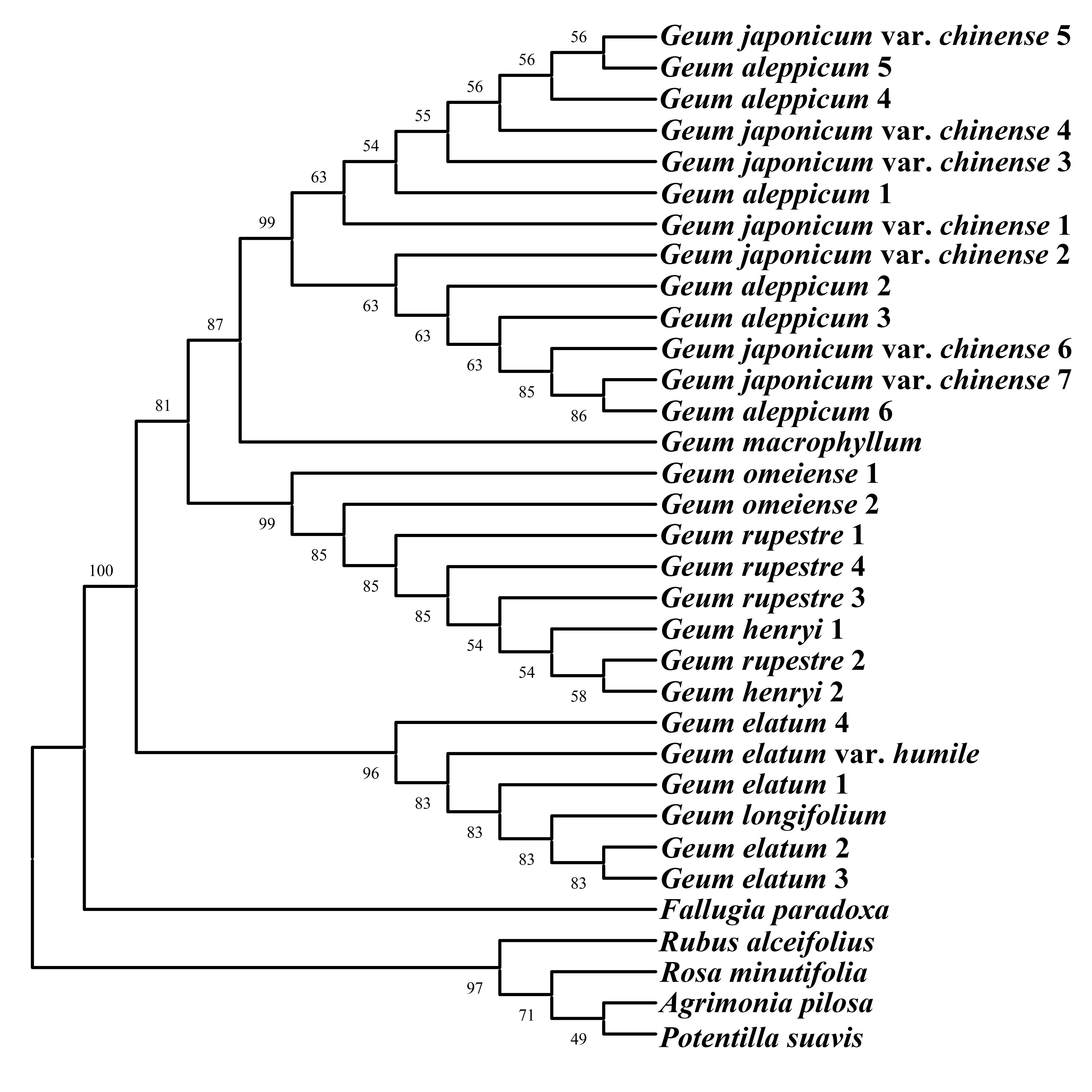

Supplement: Supplementary file 1 [file DataSheet1.zip › Supplementary Material/Figure S15.jpg]

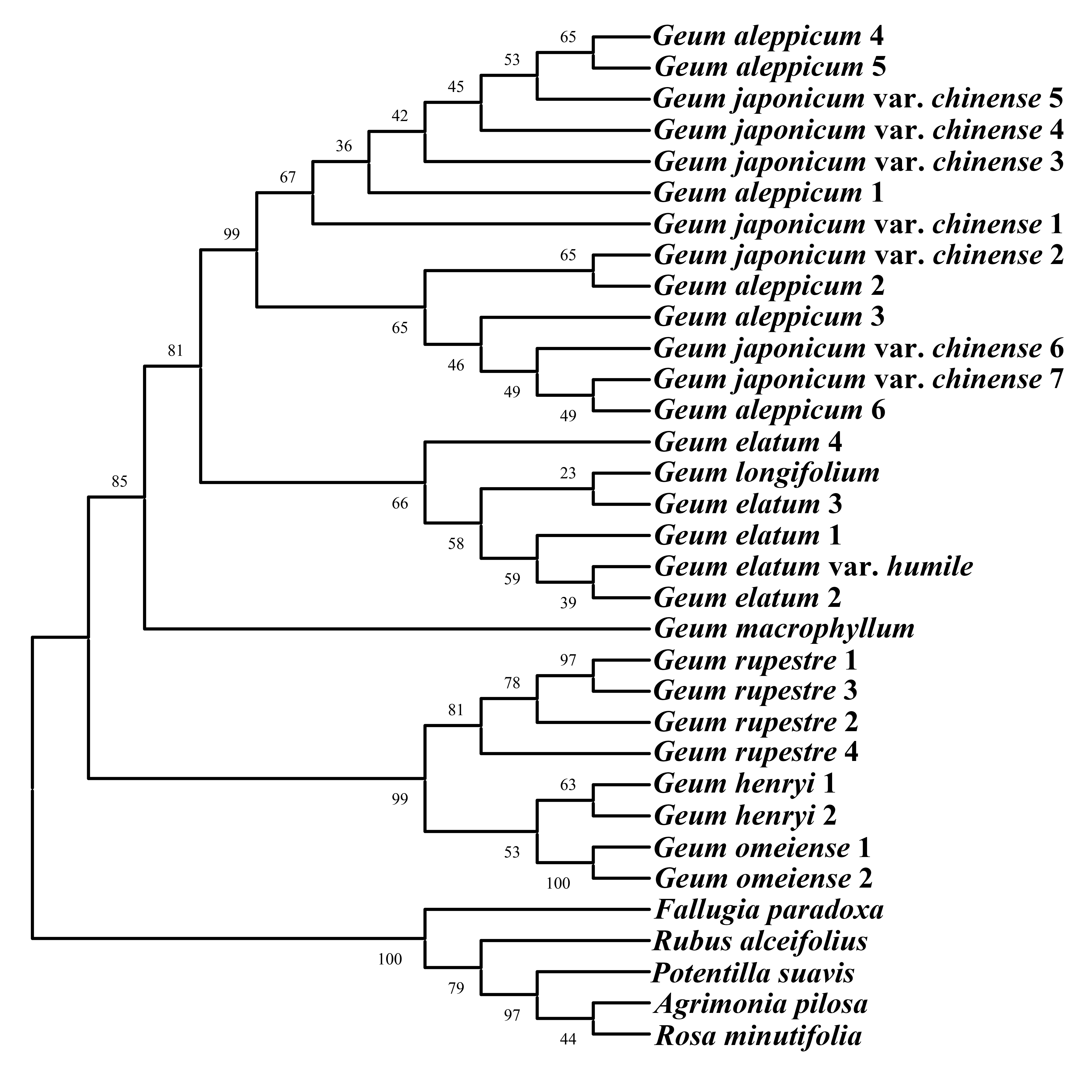

Supplement: Supplementary file 1 [file DataSheet1.zip › Supplementary Material/Figure S16.jpg]

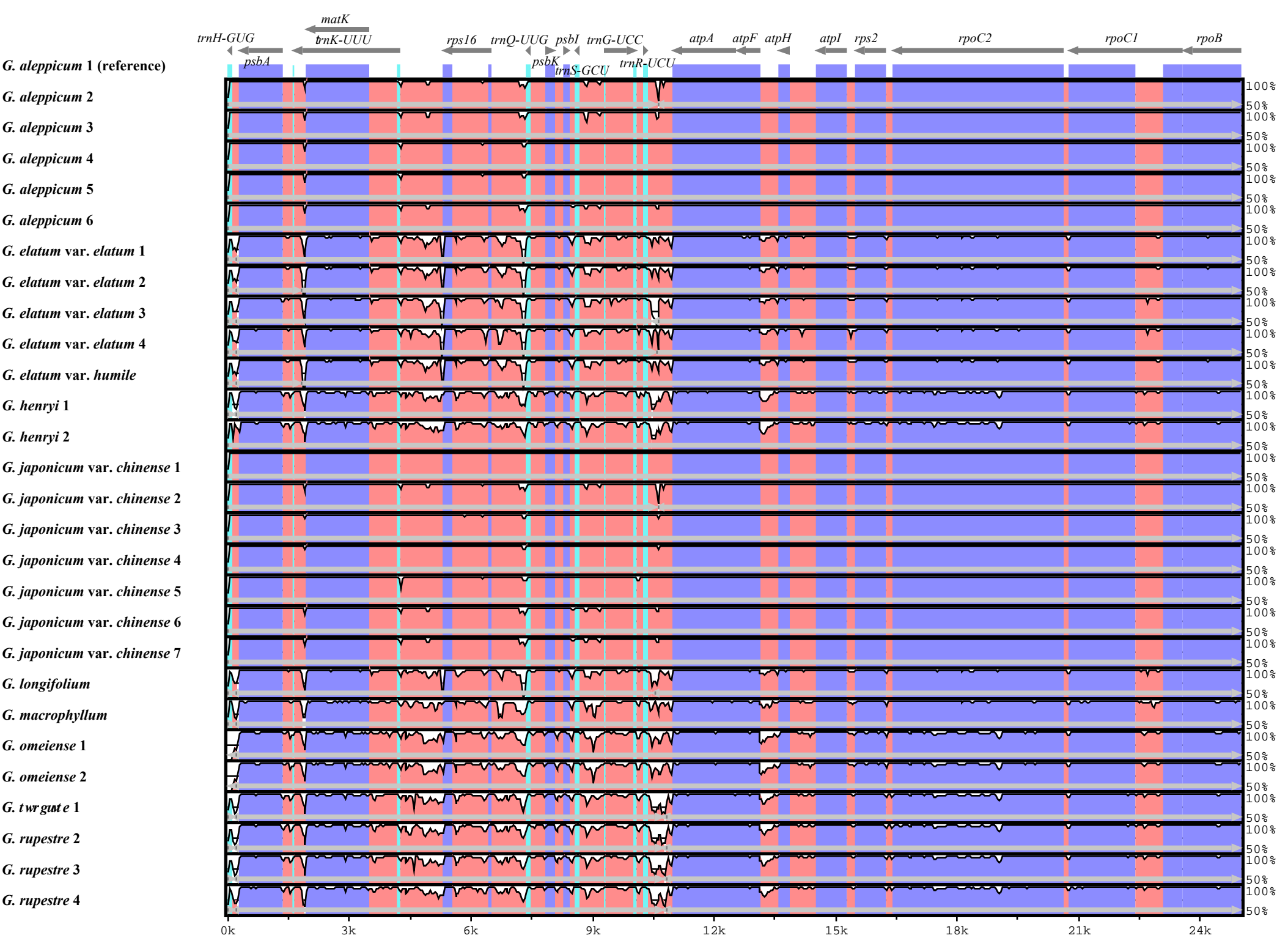

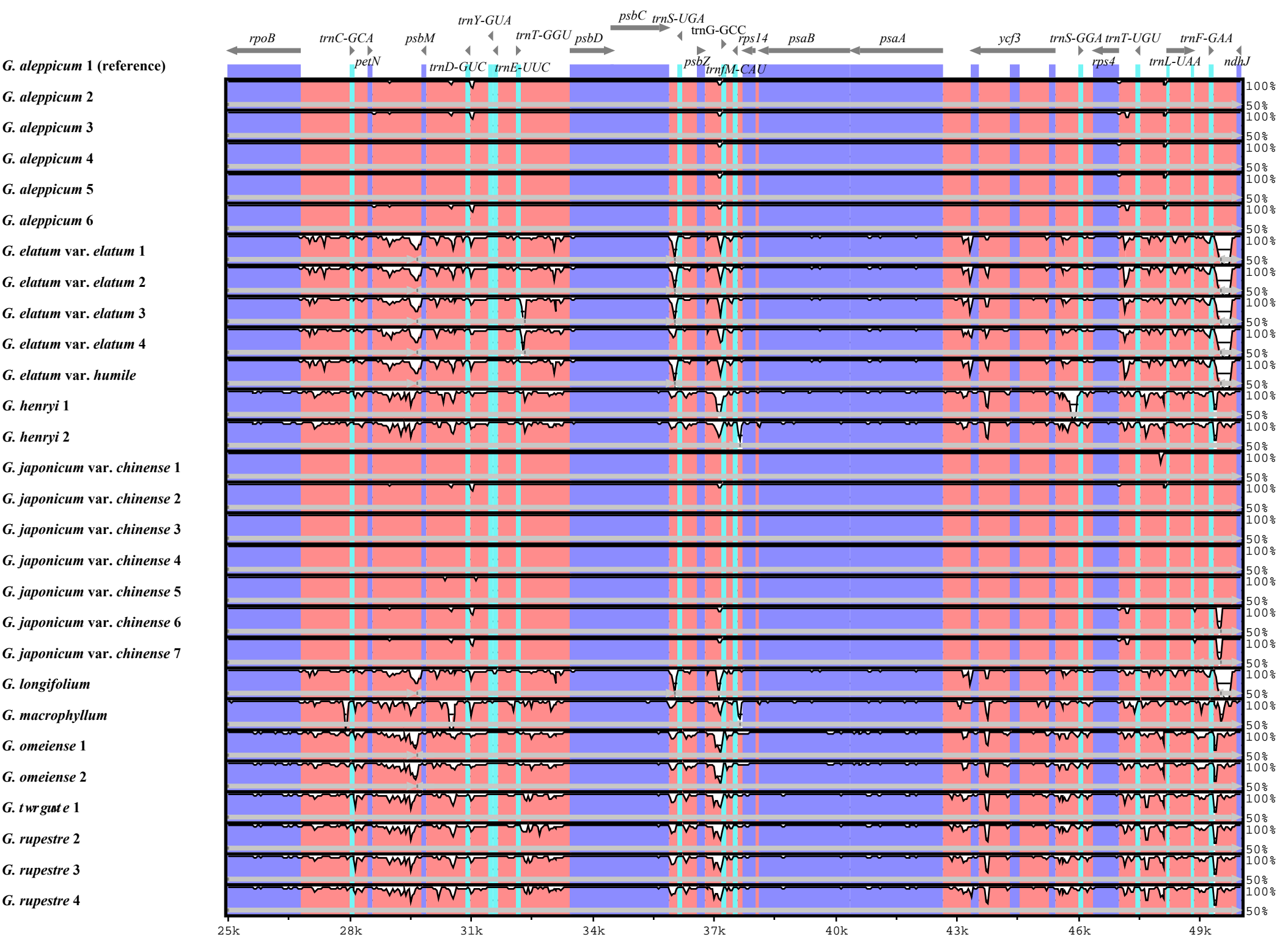

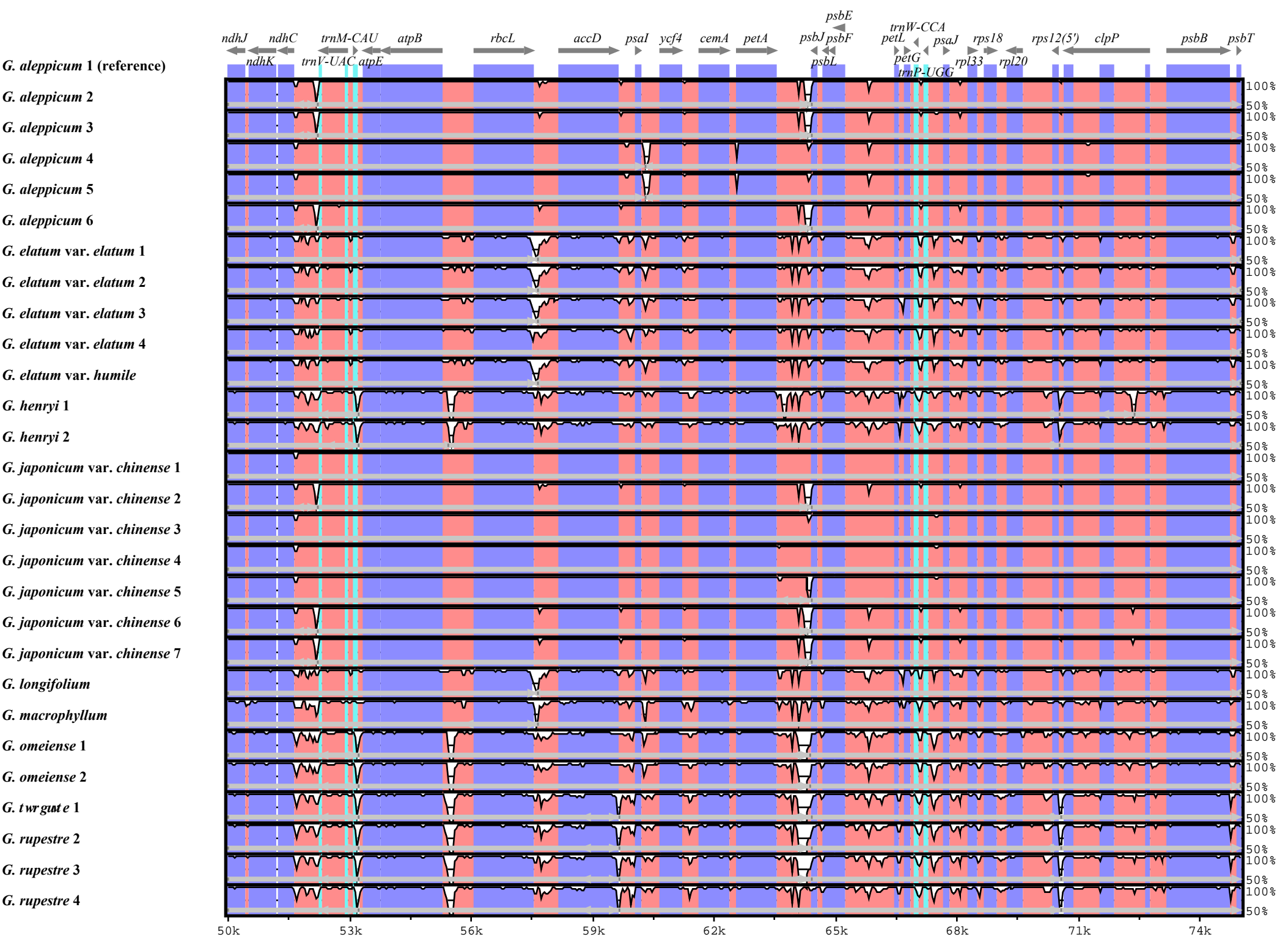

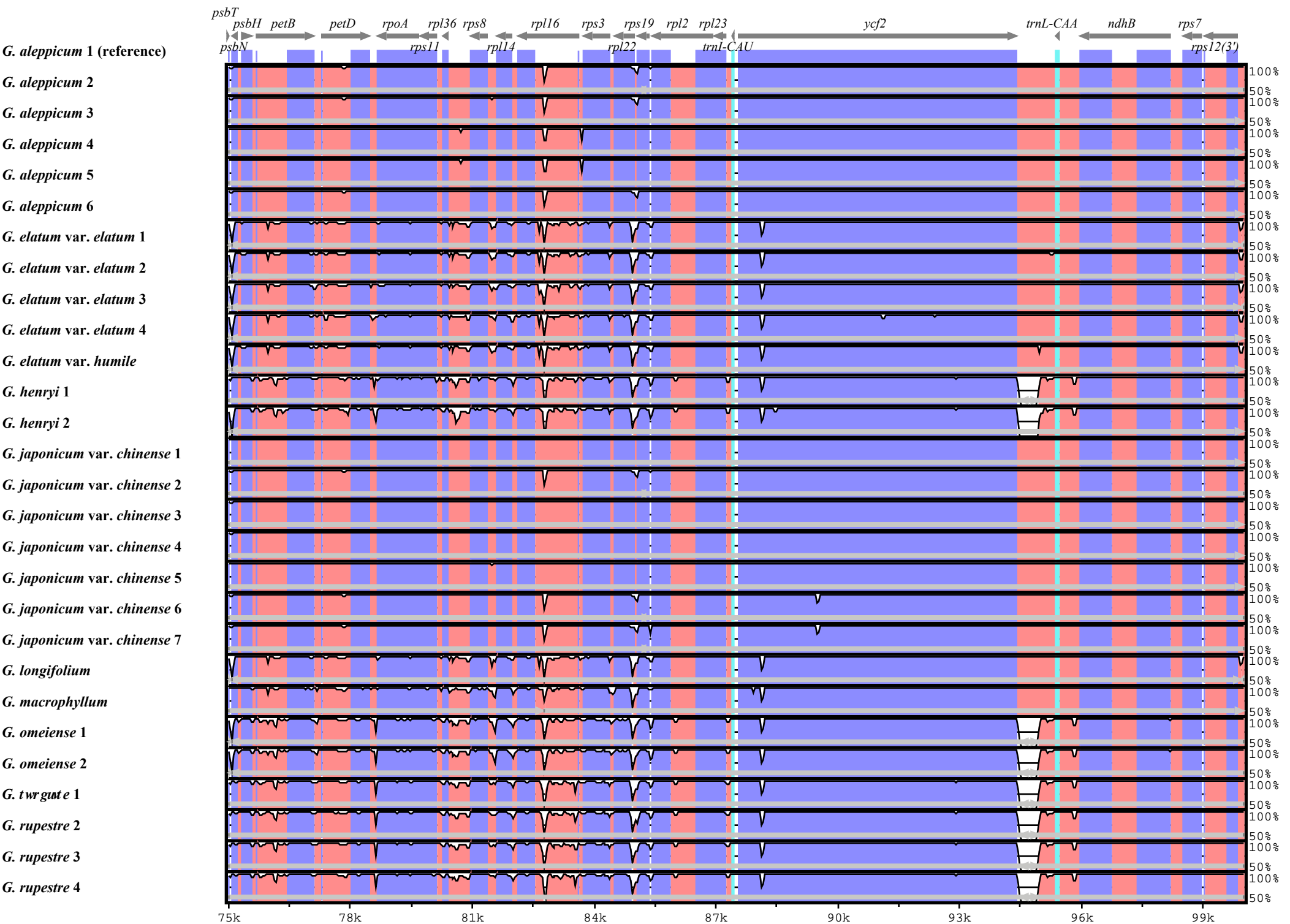

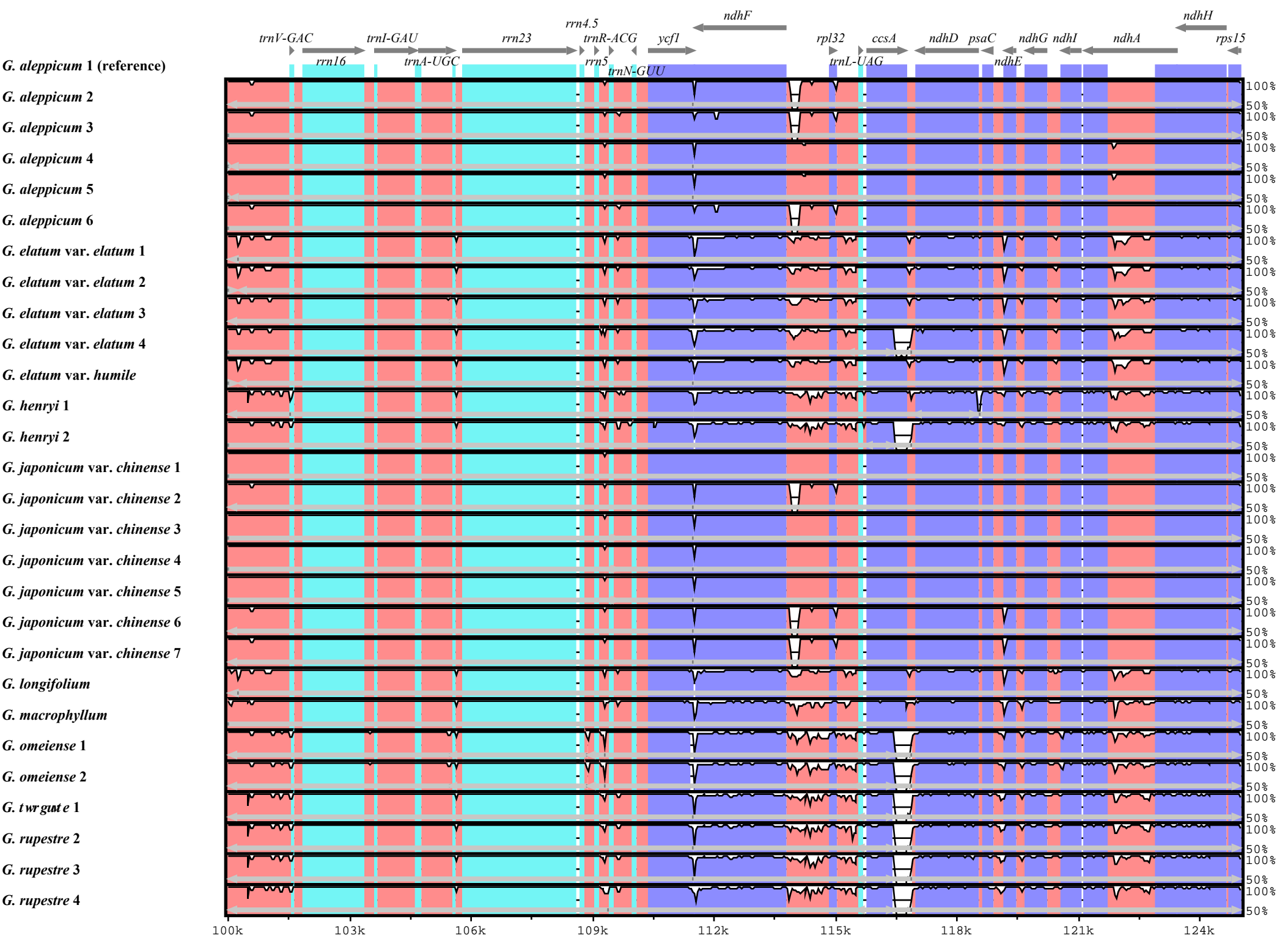

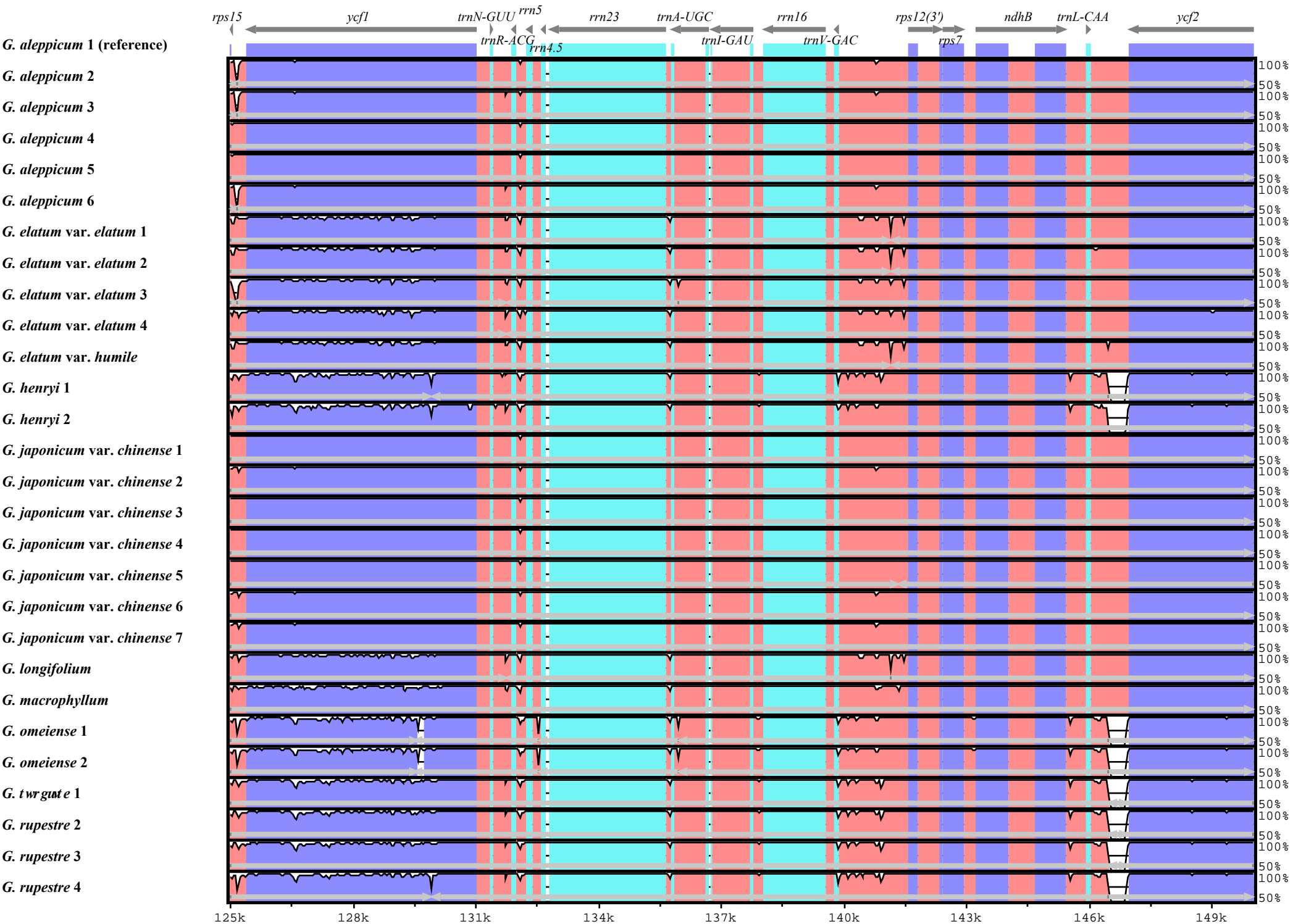

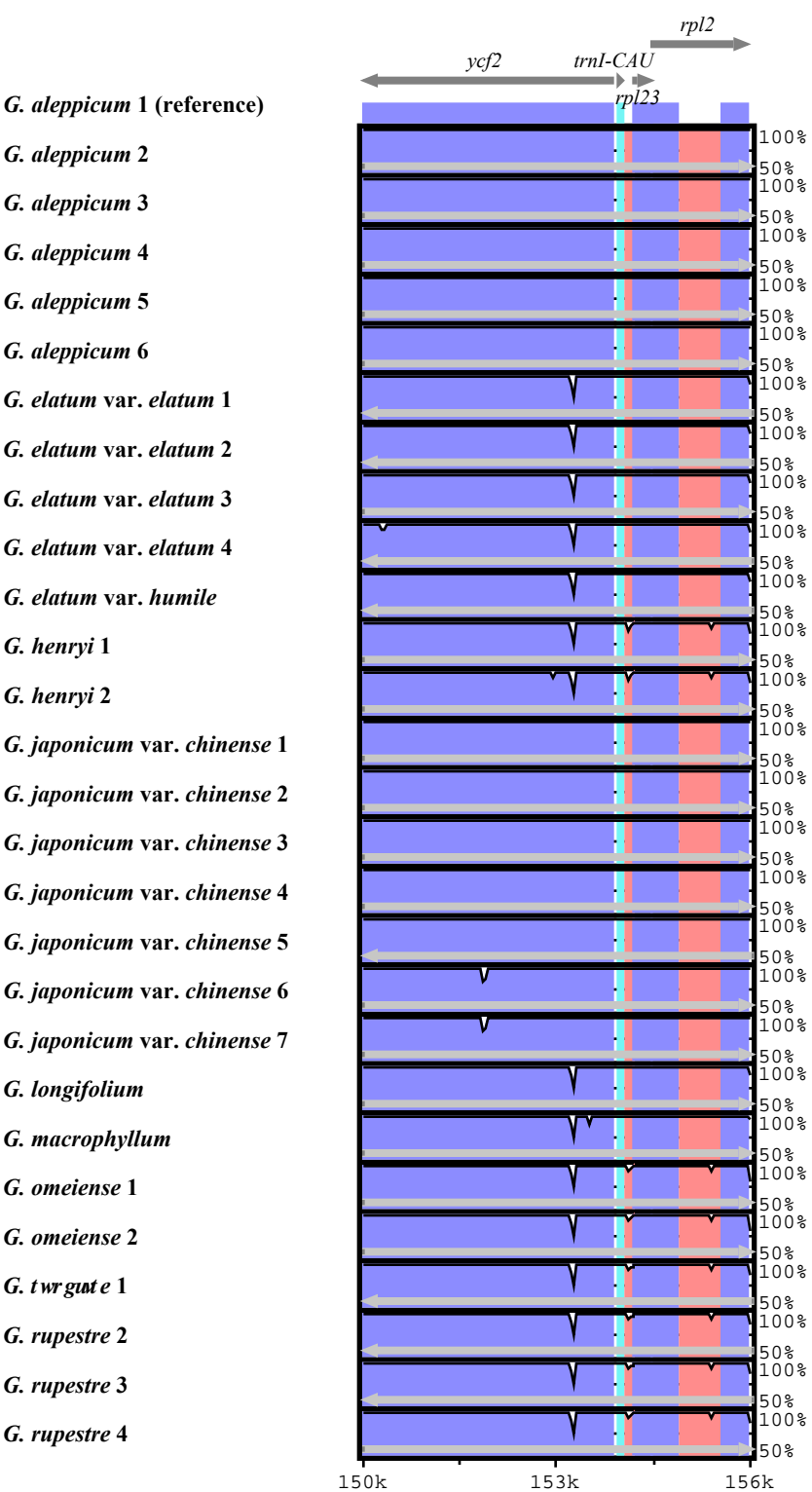

Supplement: Supplementary file 1 [file DataSheet1.zip › Supplementary Material/Figure S2.pdf]

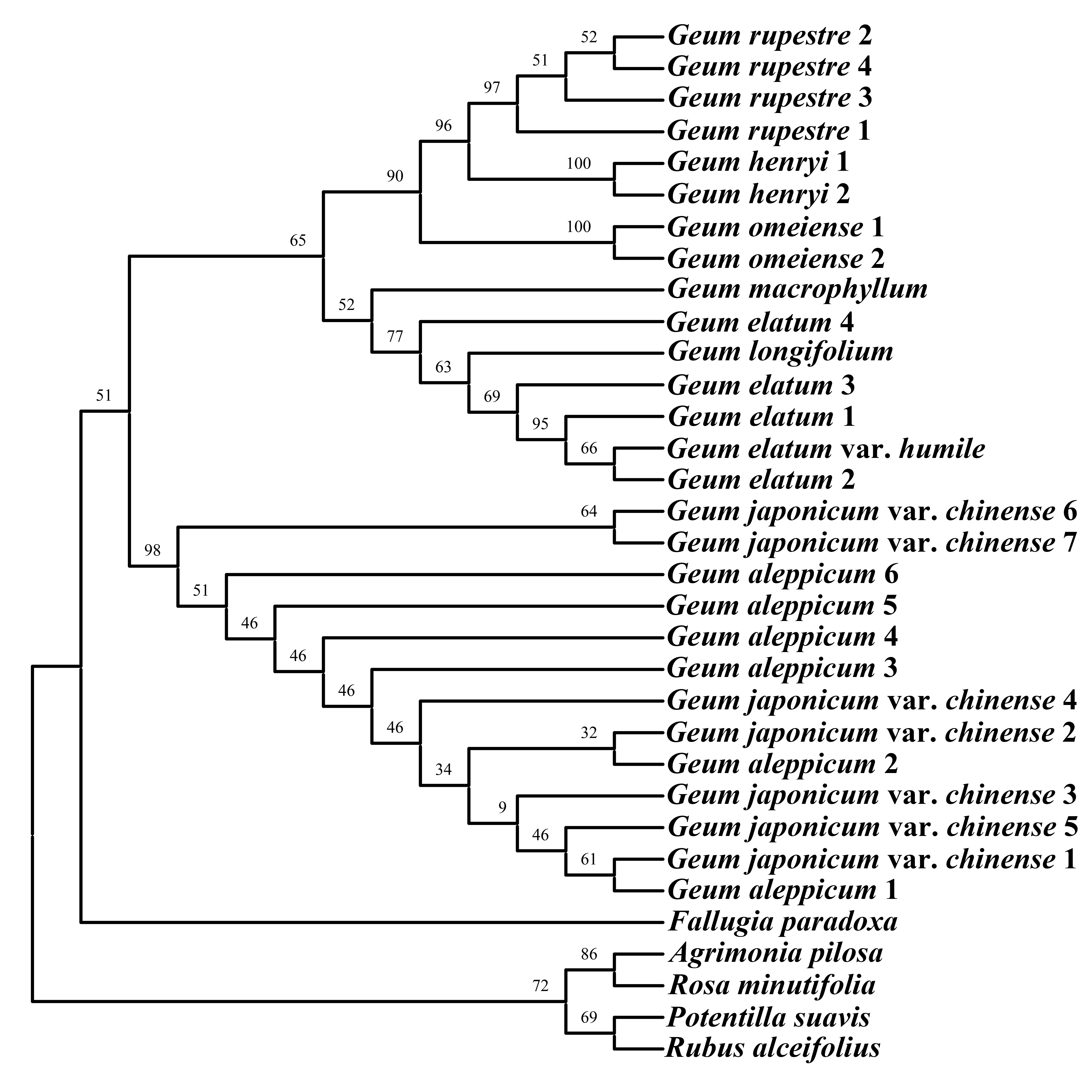

Supplement: Supplementary file 1 [file DataSheet1.zip › Supplementary Material/Figure S3.jpg]

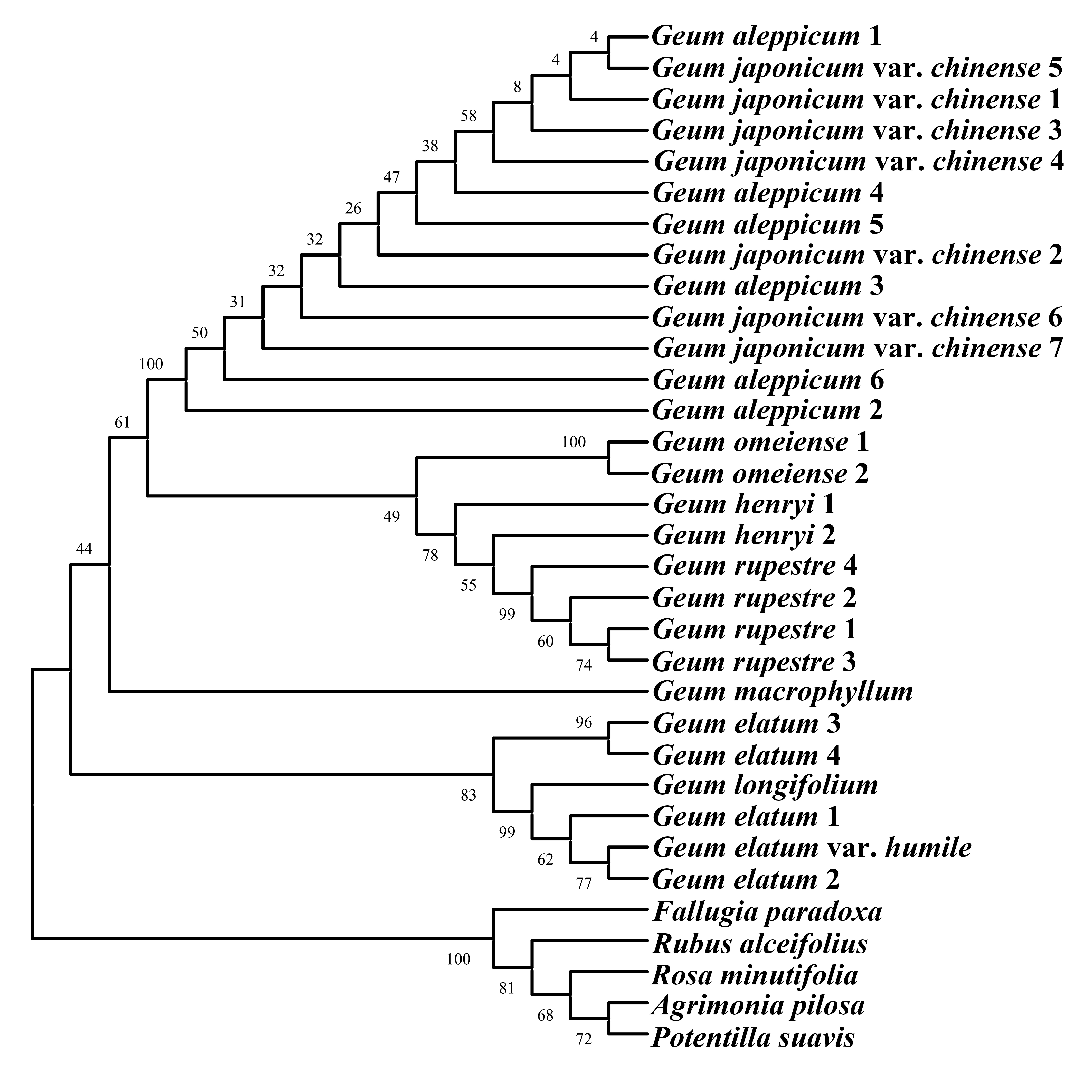

Supplement: Supplementary file 1 [file DataSheet1.zip › Supplementary Material/Figure S4.jpg]

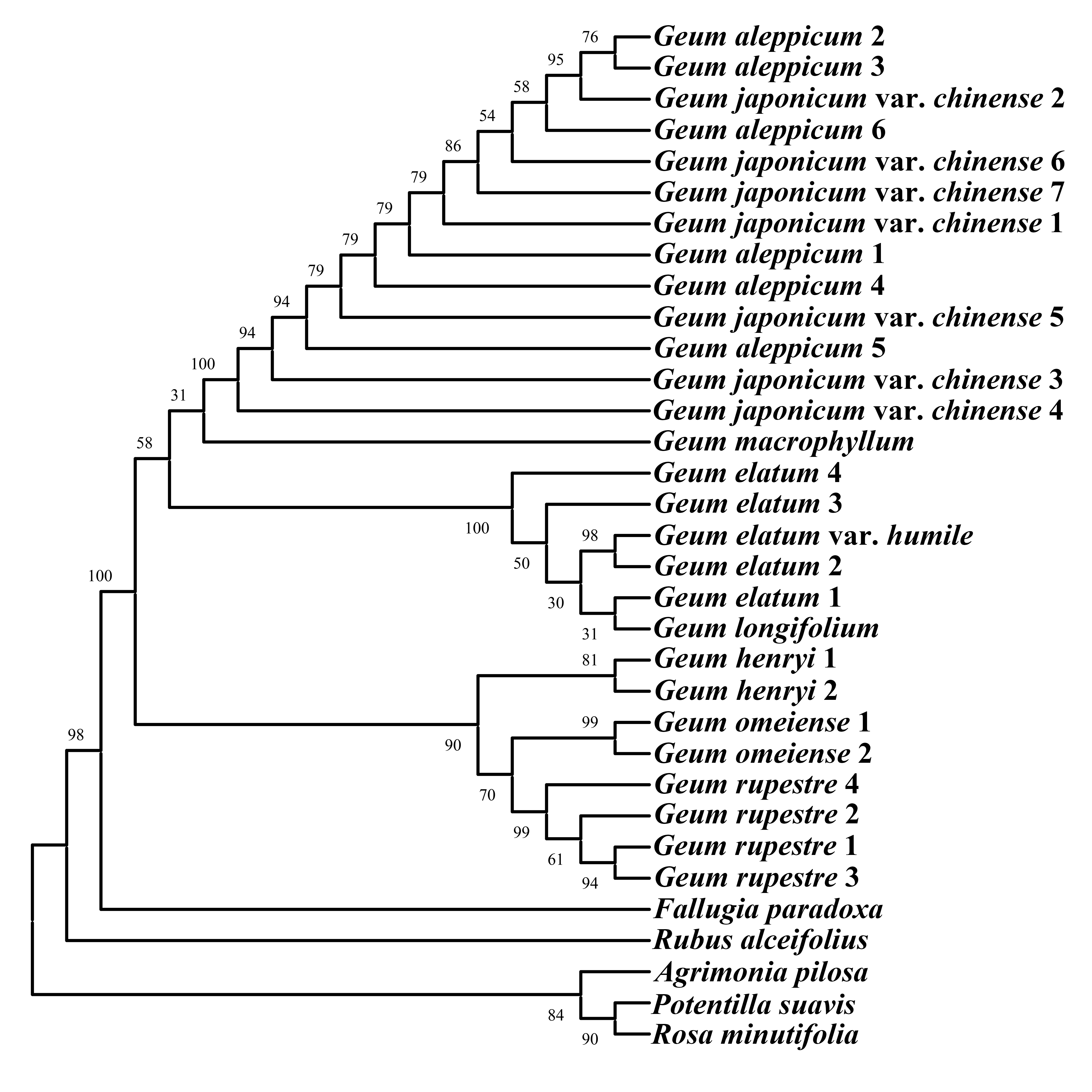

Supplement: Supplementary file 1 [file DataSheet1.zip › Supplementary Material/Figure S5.jpg]

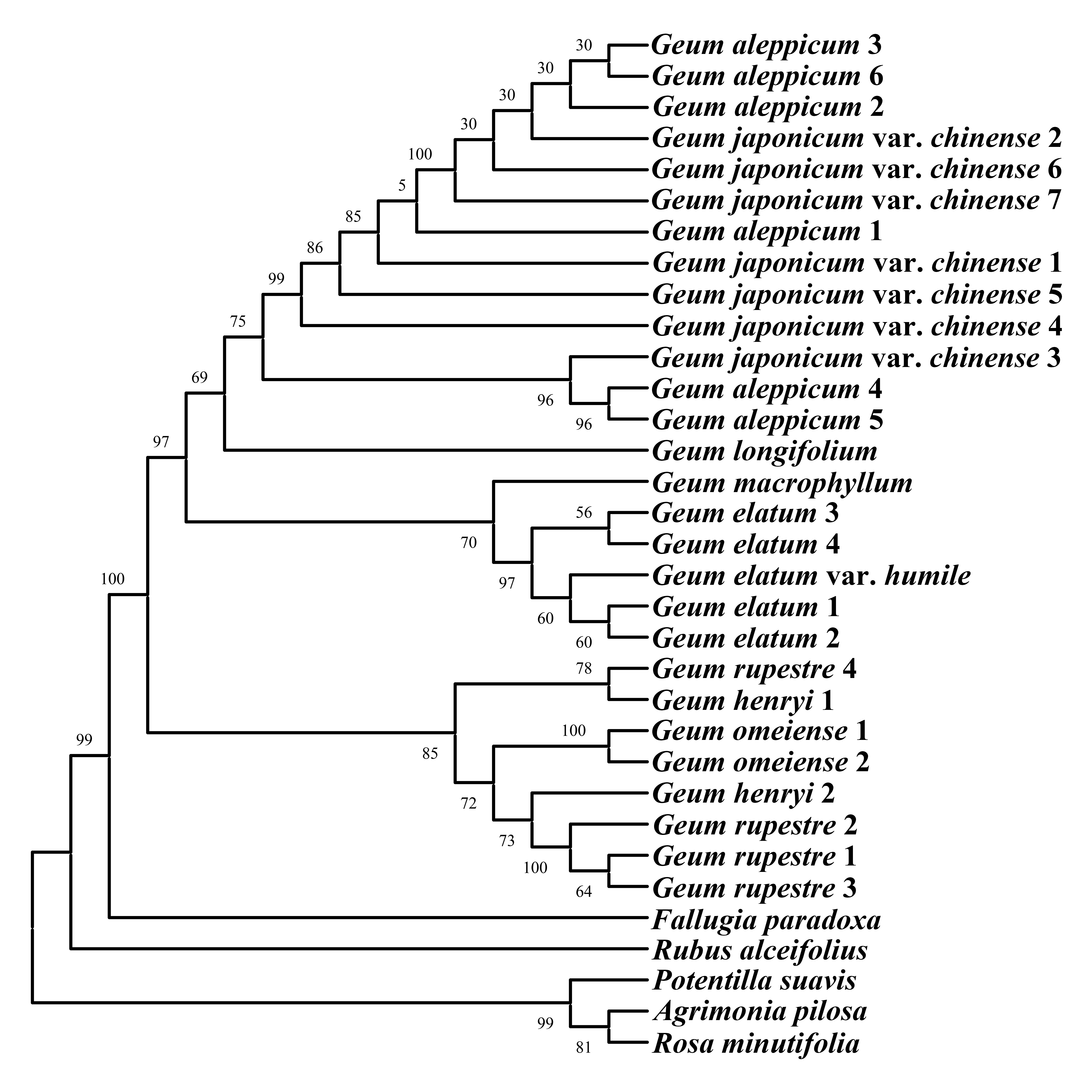

Supplement: Supplementary file 1 [file DataSheet1.zip › Supplementary Material/Figure S6.jpg]

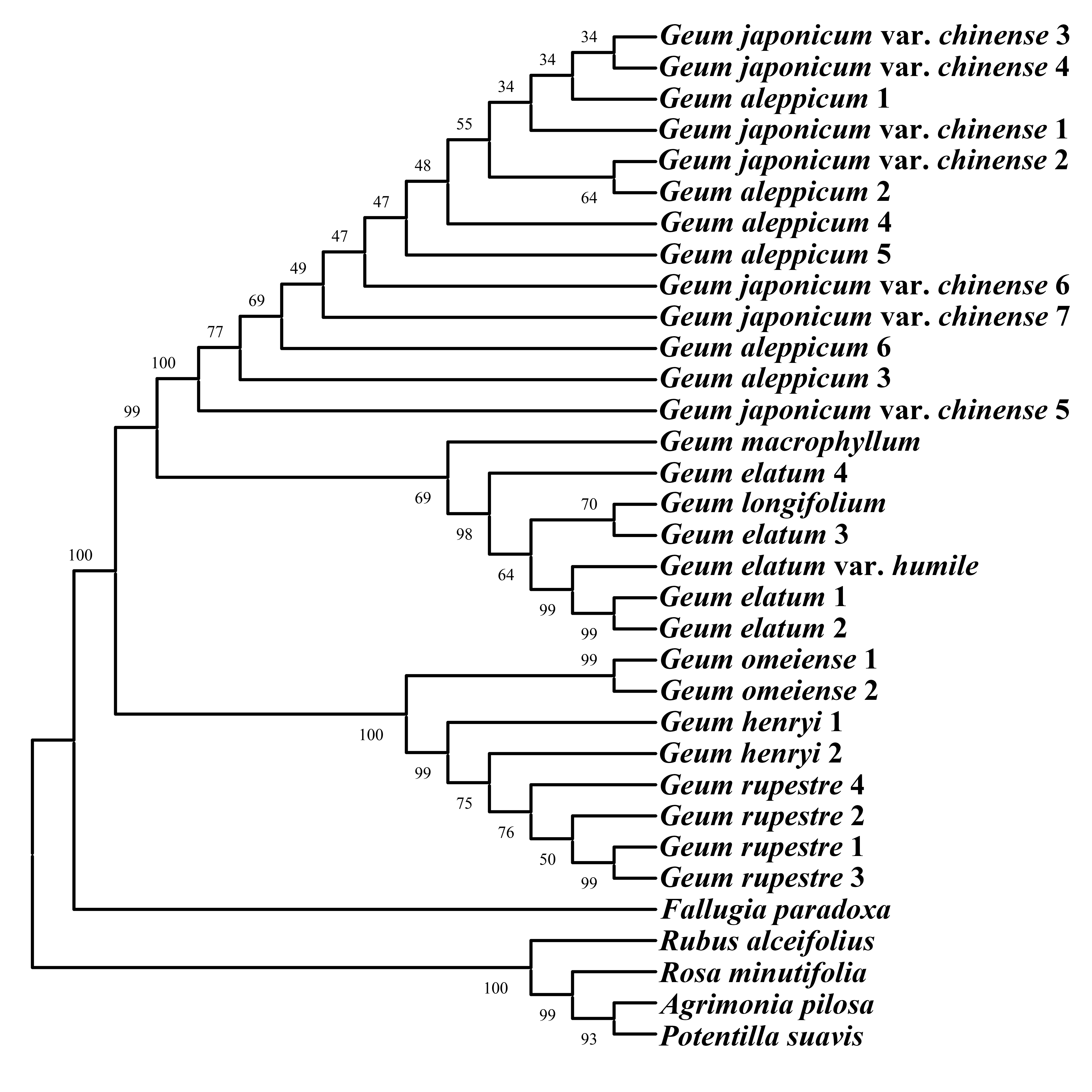

Supplement: Supplementary file 1 [file DataSheet1.zip › Supplementary Material/Figure S7.jpg]

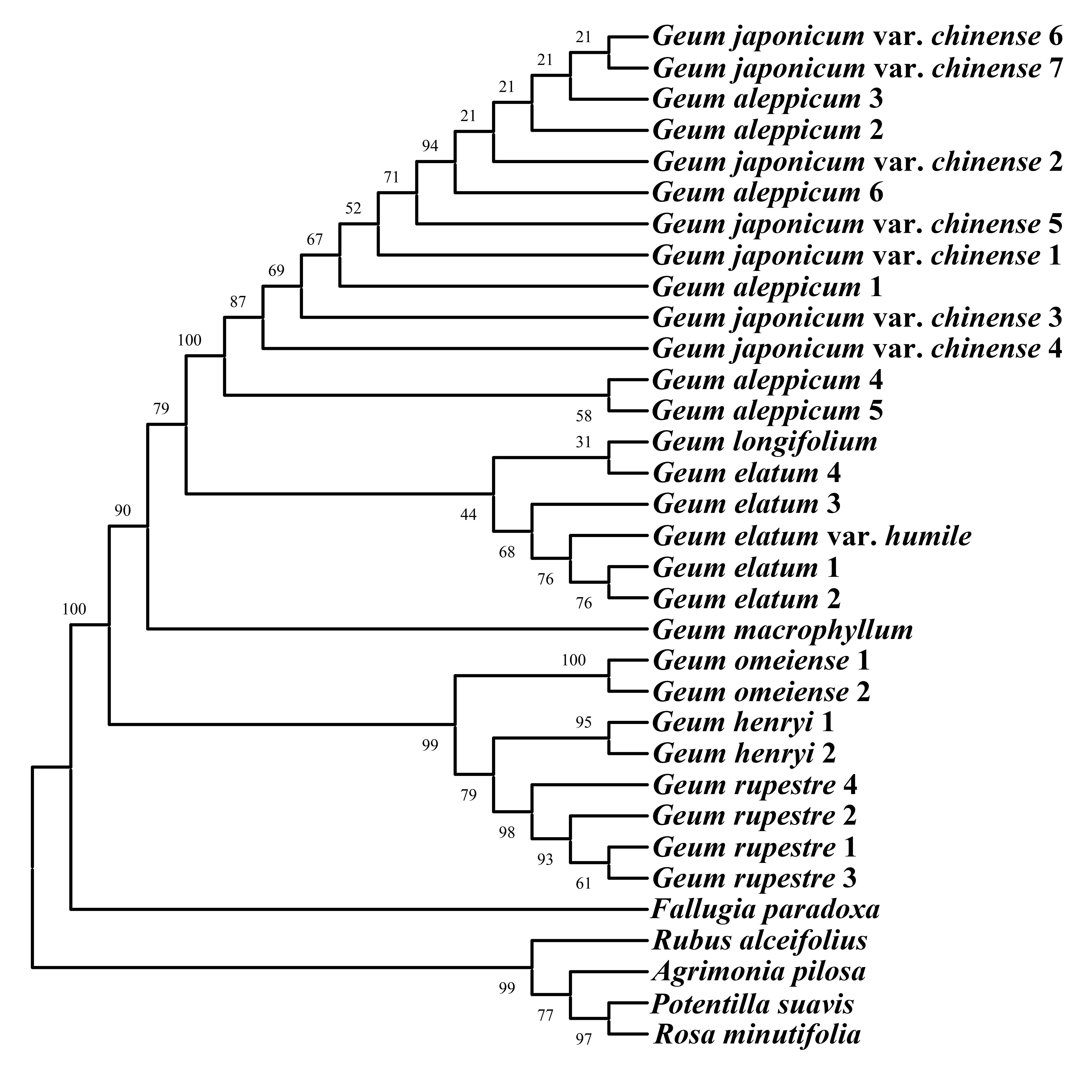

Supplement: Supplementary file 1 [file DataSheet1.zip › Supplementary Material/Figure S8.jpg]

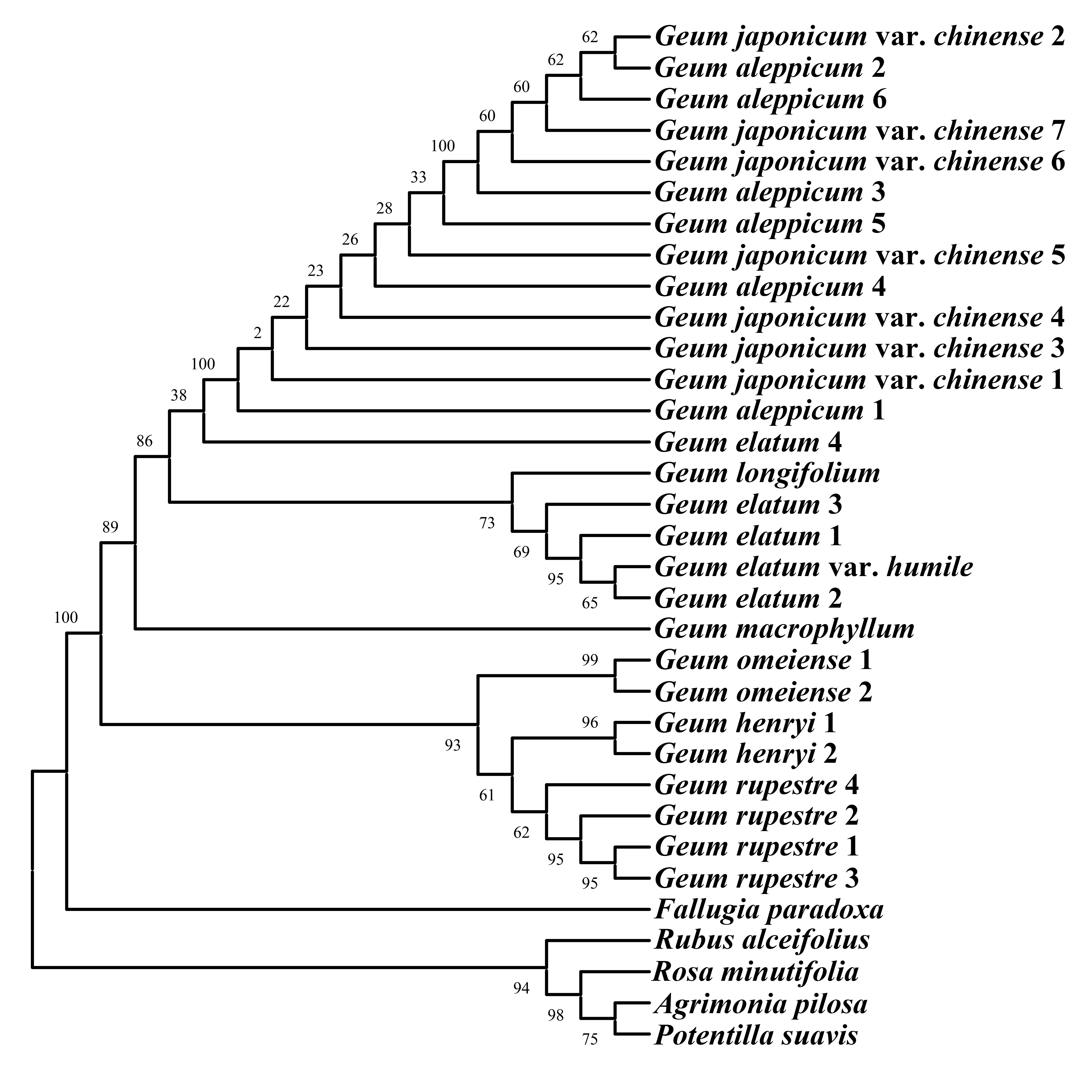

Supplement: Supplementary file 1 [file DataSheet1.zip › Supplementary Material/Figure S9.jpg]
