# Supplementary material for: Comparative analyses of chloroplast genomes in Geum species: insights into genome characteristics, phylogenomic implications, and adaptive evolution
Source: Front Plant Sci. 2025 Dec 4;16:1713809. doi: 10.3389/fpls.2025.1713809 (PMC12809601; doi:10.3389/fpls.2025.1713809)
Supplement: Supplementary file 1 [file DataSheet1.zip › Supplementary Material/Table S1.doc]

Table S1. The GenBank accession numbers of all the 37 chloroplast genome sequences used for phylogenetic analyses.

| Order | Species | GenBank accession numbers |
| --- | --- | --- |
| 1 | *Geum aleppicum* Jacq. 1 | OK509085 |
| 2 | *Geum aleppicum* Jacq. 2 | OM461318 |
| 3 | *Geum aleppicum* Jacq. 3 | PX414088 |
| 4 | *Geum aleppicum* Jacq. 4 | PX414089 |
| 5 | *Geum aleppicum* Jacq. 5 | PX414090 |
| 6 | *Geum aleppicum* Jacq. 6 | PX414091 |
| 7 | *Geum elatum* Wall. ex G.Don var. *elatum* 1 | PX414092 |
| 8 | *Geum elatum* Wall. ex G.Don var. *elatum* 2 | MT982432 |
| 9 | *Geum elatum* Wall. ex G.Don var. *elatum* 3 | PX414093 |
| 10 | *Geum elatum* Wall. ex G.Don var. *elatum* 4 | PX414094 |
| 11 | *Geum elatum* Wall. ex G.Don var. *elatum* 5 | KY419976 |
| 12 | *Geum elatum* Wall. ex G.Don var. *humile* (Royle) Hook.f. | PX414095 |
| 13 | *Geum henryi* (Batalin) Smedmark 1 | PX414096 |
| 14 | *Geum henryi* (Batalin) Smedmark 2 | PX414097 |
| 15 | *Geum japonicum* Thunb. var. *chinense* F.Bolle 1 | MW770453 |
| 16 | *Geum japonicum* Thunb. var. *chinense* F.Bolle 2 | MW770454 |
| 17 | *Geum japonicum* Thunb. var. *chinense* F.Bolle 3 | PX414098 |
| 18 | *Geum japonicum* Thunb. var. *chinense* F.Bolle 4 | PX414099 |
| 19 | *Geum japonicum* Thunb. var. *chinense* F.Bolle 5 | PX414100 |
| 20 | *Geum japonicum* Thunb. var. *chinense* F.Bolle 6 | PX414101 |
| 21 | *Geum japonicum* Thunb. var. *chinense* F.Bolle 7 | PX414102 |
| 22 | *Geum longifolium* (Maxim.) Smedmark | OP161499 |
| 23 | *Geum macrophyllum* Willd. | MT774132 |
| 24 | *Geum omeiense* (T.C.Ku) Smedmark 1 | PX414103 |
| 25 | *Geum omeiense* (T.C.Ku) Smedmark 2 | PX414104 |
| 26 | *Geum rupestre* (T.T.Yü & C.L.Li) Smedmark 1 | MZ151697 |
| 27 | *Geum rupestre* (T.T.Yü & C.L.Li) Smedmark 2 | MG262388 |
| 28 | *Geum rupestre* (T.T.Yü & C.L.Li) Smedmark 3 | ON873898 |
| 29 | *Geum rupestre* (T.T.Yü & C.L.Li) Smedmark 4 | ON873891 |
| 30 | *Geum triflorum* Pursh | KY419977 |
| 31 | *Geum urbanum* L. 1 | OX327019 |
| 32 | *Geum urbanum* L. 2 | ON556622 |
| 33 | *Agrimonia pilosa* Ledeb. | MT040192 |
| 34 | *Fallugia paradoxa* (D.Don ex Tilloch & Taylor) Endl. ex Torr. | KY419999 |
| 35 | *Potentilla suavis* Soják | MT114190 |
| 36 | *Rosa minutifolia* Engelm. | MT755634 |
| 37 | *Rubus alceifolius* Poir. | PP566745 |
